# The impact of schistosomiasis on the Global Disease Burden: a systematic analysis based on the 2021 Global Burden of Disease study

**DOI:** 10.1051/parasite/2025005

**Published:** 2025-02-21

**Authors:** Zhangzhou Shen, Houqiang Luo

**Affiliations:** 1 Hubei Key Laboratory for Kidney Disease Pathogenesis and Intervention, Hubei Polytechnic University, Medical School Huangshi 435003 Hubei China; 2 College of Animal Science, Wenzhou Vocational College of Science and Technology Wenzhou 325006 Zhejiang China

**Keywords:** Schistosomiasis, Global burden, Age-standardized rates, EAPC

## Abstract

Schistosomiasis is a neglected tropical disease that causes a significant global burden. The aim of this study was to report the latest estimates of the global, regional, and national schistosomiasis disease burden and forecast changes in schistosomiasis-related disease burden. This work was based on the 2021 Global Burden of Disease (GBD) study. We analyzed the schistosomiasis data by sex, age in years, and Socio-Demographic Index (SDI) region and country, using Age-Standardized Rates (ASR) for comparisons among different groups. The Estimated Annual Percent Changes (EAPC) analysis was used to evaluate the temporal trend of the disease burden, and the Differential Autoregressive Integrated Moving Average (ARIMA) and Exponential Smoothing (ES) models were used to predict the disease burden from 2022 to 2046. In the GBD 2021 study, it was reported that compared to 1990, the number of deaths has decreased by 74,350, the prevalence number has increased by 1,482,260, and Disability-Adjusted Life Years (DALYs) have decreased by 1,770,436. Additionally, the age-standardized mortality rate (ASMR) has decreased by 0.31 per 100,000 people, with an EAPC of −0.353 (95% CI: −0.361 to −0.344). Similarly, the age-standardized DALYs rate (ASDR) has decreased by 15.45 per 100,000 people (EAPC: −1.56, 95% CI: −1.78 to −1.34), and the age-standardized prevalence rate (ASPR) has decreased by 559.64 per 100,000 people (EAPC: −0.63, 95% CI: −0.95 to −0.31). The regions and countries with the highest disease burden are mostly concentrated in Africa. Despite a general decline in global schistosomiasis burden indicators, the burden of disease has actually increased in high SDI areas. The ARIMA and ES models forecast results show that female mortality and ASMR will decline in the next 25 years, while male mortality and ASMR will remain stable, and other disease indicators will continue to decline. The global schistosomiasis burden has significantly decreased over the past 30 years, but it remains high in African regions and countries, as well as low-SDI areas. Effective cooperation among countries should be strengthened to improve the disease burden in high-burden areas and countries.

## Abstract (fr)

La schistosomiase est une maladie tropicale négligée qui représente un fardeau mondial important. L’objectif de cette étude est de rapporter les dernières estimations de la charge mondiale, régionale et nationale de la schistosomiase et de prévoir l’évolution de la charge de morbidité liée à la schistosomiase. Cette étude est basée sur l’étude sur la Charge Mondiale de Morbidité (GBD) de 2021. Nous avons analysé les données sur la schistosomiase par sexe, âge en années et indice sociodémographique (SDI) région et pays, en utilisant les taux standardisés selon l’âge (ASR) pour les comparaisons entre différents groupes. L’analyse des variations annuelles estimées en pourcentage (EAPC) a été utilisée pour évaluer la tendance temporelle de la charge de morbidité, et les modèles de moyenne mobile intégrée autorégressive différentielle (ARIMA) et de lissage exponentiel (ES) ont été utilisés pour prédire la charge de morbidité de 2022 à 2046. Dans l’étude GBD 2021, il est rapporté que par rapport à 1990, le nombre de décès a diminué de 74 350, le nombre de prévalence a augmenté de 1 482 260, les AVCI (années de vie ajustées sur l’incapacité) ont diminué de 1 770 436. De plus, le taux de mortalité standardisé selon l’âge (ASMR) a diminué de 0,31 pour 100 000 personnes, avec une variation annuelle estimée en pourcentage (EAPC) de −0,353 (IC à 95 % : −0,361 à −0,344). De même, le taux d’AVCI standardisé selon l’âge (ASDR) a diminué de 15,45 pour 100 000 personnes (EAPC : −1,56, IC à 95 % : −1,78 à −1,34), et le taux de prévalence standardisé selon l’âge (ASPR) a diminué de 559,64 pour 100 000 personnes (EAPC : −0,63, IC à 95 % : −0,95 à −0,31). Les régions et les pays où la charge de morbidité est la plus élevée sont principalement concentrés en Afrique. Malgré une baisse générale des indicateurs de la charge de morbidité de la schistosomiase à l’échelle mondiale, la charge de morbidité a en fait augmenté dans les zones à fort SDI. Les résultats des prévisions des modèles ARIMA et ES montrent que la mortalité féminine et l’ASMR diminueront au cours des 25 prochaines années, tandis que la mortalité masculine et l’ASMR resteront stables et que les autres indicateurs de maladie continueront de baisser. La charge mondiale de morbidité due à la schistosomiase a considérablement diminué au cours des 30 dernières années, mais elle reste élevée dans les régions et pays africains, ainsi que dans les zones à indice sociodémographique bas. Il convient de renforcer la coopération entre les pays pour alléger la charge de morbidité dans les zones et pays à forte prévalence.

## Abbreviations

NTD, Neglected Tropical Disease; ASR, Age-Standardized Rates; EAPC, Estimated Annual Percent Changes; ASMR, Age-standardized Mortality Rate; ASDR, Age-Standardized DALY Rate; ASPR, Age-Standardized Prevalence Rate; SDI, Socio-Demographic Index; UI, Uncertainty Interval, ARIMA, Autoregressive Integrated Moving Average; ES, Exponential Smoothing.

## Introduction

Schistosomiasis, a parasitic infection caused by the larvae of blood flukes and transmitted through the human vein system, has long posed a significant threat to global public health [10, 11, 32]. The World Health Organization (WHO) has classified it as one of the Neglected Tropical Diseases (NTDs), with its high incidence rate and widespread geographic distribution, making its global disease burden particularly significant [8]. Schistosomiasis mainly spreads in countries and regions in Asia, Africa, and Latin America, affecting residents in impoverished areas and being difficult to eradicate due to its complex transmission chain and wide host range [1, 3, 4].

Schistosomiasis poses a significant threat to human health, with early typical clinical symptoms including rash, cough, fever, abdominal pain, and diarrhea [7, 13], while the late stage may lead to liver and spleen enlargement, liver cirrhosis, ascites, and even hepatic coma, gastrointestinal bleeding, and death [12, 13, 18, 19]. Moreover, schistosomiasis has a particularly high impact on the health of women and children, with women’s health potentially affected by the disease, leading to infertility or miscarriage [5], while children’s health may be affected, leading to stunted growth and, in severe cases, short stature [7]. It is estimated that schistosomiasis causes a global disease burden of approximately 3.31 million disability-adjusted life years (DALYs), which highlights the significant consumption of global public health resources by the disease [15]. Schistosomiasis not only affects patients’ physical health, but also has a profound impact on their social and economic status, especially in poor areas with limited medical resources, where the disease often becomes one of the main causes of poverty and re-poverty [26].

In summary, the global disease burden of schistosomiasis is very high, causing significant impact on individuals’ health, social and economic status, and public health, and is an urgent public health issue that needs to be addressed [25]. Therefore, it is essential to conduct in-depth research on the global disease burden of schistosomiasis. This study aimed to provide important references for analyzing the epidemiological characteristics and patterns of schistosomiasis worldwide, evaluating its disease burden, and formulating effective control strategies. Therefore, the primary objective of this study was to analyze and forecast trends in schistosomiasis deaths, DALYs, and prevalence from 2020 to 2045, using available data and advanced predictive models.

## Materials and methods

### Study population and data sources

This study utilized data from the GBD website (https://vizhub.healthdata.org/gbd-results/) [6] from 1990 to 2021 on global schistosomiasis-related death number, DALYs, and prevalence number and corresponding age-standardized rates (ASRs), including Age-Standardized Mortality Rate (ASMR), Age-Standardized DALY Rate (ASDR), and Age-Standardized Prevalence Rate (ASPR). The GBD study is a systematic investigation that assessed the impact of 370 diseases and risk factors on health across sex, age, and Socio-Demographic Index (SDI), and 204 countries and 54 GBD regions. The study population was divided into 20 groups; it also included all SDI regions (high SDI, middle-high SDI, middle-high SDI, middle-low SDI, and low SDI), 54 GBD regions (only 41 GBD regions reported data values), and 204 countries and regions (only 70 countries and regions reported data values for deaths and DALYs).

### Autoregressive integrated moving average and exponential smoothing models

In the disease burden forecasting section, this study employed the Differential Autoregressive Integrated Moving Average (ARIMA) model [35], which is a widely used statistical forecasting method for time series prediction problems. The ARIMA model can capture different types of time series features by adjusting the parameters (p, d, q). By analyzing the historical data of schistosomiasis, the study predicted the schistosomiasis-related death number, DALYs, prevalence number, ASMR, ASDR and ASPR from 2022 to 2046. Based on schistosomiasis data from 1990–2020, this study employs the Exponential Smoothing (ES) model to forecast trends in schistosomiasis disease burden from 2020 to 2045 [36]. In applying the ES model, the smoothing coefficient (smoothing parameter) needs to be adjusted to balance the weight of historical data and new data, and the optimal smoothing coefficient is selected through cross-validation or grid search methods. ES is a commonly used time series forecasting method that predicts the future 25-year trend of schistosomiasis by weighted averaging of historical data. Through the above two forecasting methods, we can determine whether the future disease burden trend of schistosomiasis is consistent.

### Statistical analysis

We explored the temporal trend of the global schistosomiasis burden from 1990 to 2021 by subtypes, including age, gender, SDI, GBD region, and country, and Estimated Annual Percent Changes (EAPC) was estimated using a linear regression model. Based on the EAPC values, hierarchical clustering analysis was conducted to evaluate the trends of disease burden changes in each GBD region and identify regions with similar changes in disease burden. The 41 GBD regions were divided into four categories: significant increase, moderate increase, stable or minor decrease, and significant decrease.

Statistical significance was considered when the *p*-value was less than 0.05. The data were constructed, organized, analyzed, and plotted using R (version 4.3.0) software.

## Results

### Disease burden caused by schistosomiasis in 2021 (based on GBD 2021 data)

**Supplementary Figure 1.**
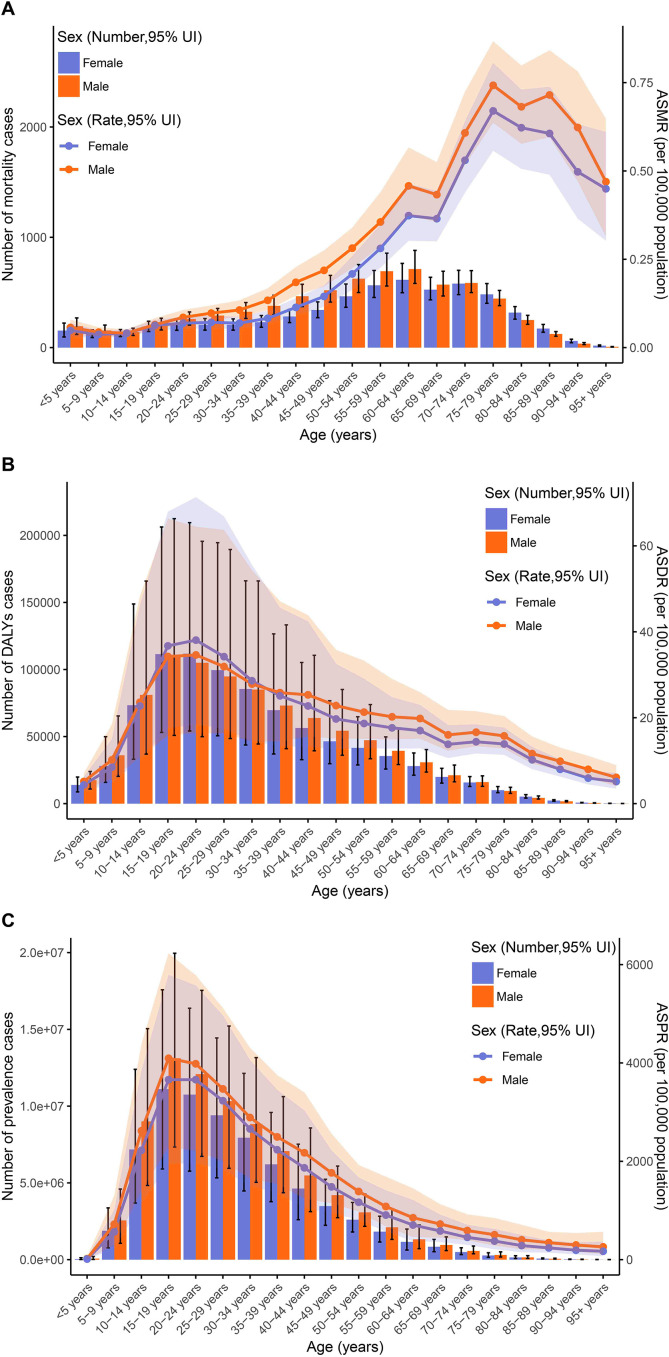
Global burden of schistosomiasis in 2021. (A) Number of deaths and ASMR. (B) Number of DALYs and ASDR. (C) Prevalence number and ASPR.

The disease burden data for schistosomiasis in 2021, presented here, are derived from the Global Burden of Disease (GBD) 2021 study and are not predictions made by our ARIMA model. According to the GBD 2021, in 2021, the number of deaths related to schistosomiasis was 12,858 people (95% uncertainty interval (UI): 11,335–14,388), with an ASMR of 0.15 per 100,000 people (95% UI: 0.14–0.17) (Table 1). The number of deaths increased with age for both men and women, peaking in the 60–64 age group, and the ASMR peaked in the 75–79 age group before gradually decreasing. Among the age groups before 70–74, the number of female deaths was generally lower than that of males (Supplementary Fig. 1A). Similarly, DALYs related to schistosomiasis were 1,746,333 (95% UI: 1,038,122–2,984,204), with an ASDR of 21.9 per 100,000 people (95% UI: 12.94–37.28) (Table 2). The DALYs and ASDR were highest among the 15–24 age group, and then decreased gradually. In the 15–34 age group, the female DALYs and ASDR were higher than those of males (Supplementary Fig. 1B). The prevalence number was 151,376,744 (95% UI: 109,062,891–198,666,396), with an ASPR of 1,914.30 per 100,000 people (95% UI: 1,378.92–2,510.85) (Table 3). The ASPR decreased gradually after the 15–19 age group.

**Table 1:** Number of deaths and age-standardized deaths rate (ASMR) attributable to schistosomiasis in 1990 and 2021, and trends from 1990 to 2021 globally.

Characteristics	Number of deaths (95% UI) in 1990	The age – standardized deaths rate/100,000 (95% UI) in 1990	Number of deaths (95% UI) in 2021	The age – standardized deaths rate/100,000 (95% UI) in 2021	EAPC (95% CI)
Global	20,293 (18,227 – 22,408)	0.46 (0.42 – 0.51)	12,858 (11,335 – 14,388)	0.15 (0.14 – 0.17)	−3.53 (−3.61 – 3.44)
**Age**					
<5 years	795 (580 – 1,084)	0.13 (0.09 – 0.17)	348 (221 – 479)	0.05 (0.03 – 0.07)	−2.66 (−2.79 – 2.53)
5–9 years	542 (400 – 714)	0.09 (0.07 – 0.12)	279 (216 – 358)	0.04 (0.03 – 0.05)	−2.21 (−2.46 – 1.95)
10–14 years	501 (401 – 626)	0.09 (0.07 – 0.12)	273 (219 – 330)	0.04 (0.03 – 0.05)	−2.43 (−2.56 – 2.3)
15–19 years	657 (549 – 779)	0.13 (0.11 – 0.15)	400 (323 – 487)	0.06 (0.05 – 0.08)	−2.39 (−2.54 – 2.25)
20–24 years	695 (578 – 824)	0.14 (0.12 – 0.17)	463 (374 – 560)	0.08 (0.06 – 0.09)	−2.15 (−2.37 – 1.94)
25–29 years	747 (629 – 890)	0.17 (0.14 – 0.2)	498 (408 – 596)	0.08 (0.07 – 0.1)	−2.24 (−2.44 – 2.04)
30–34 years	780 (652 – 917)	0.2 (0.17 – 0.24)	532 (442 – 650)	0.09 (0.07 – 0.11)	−2.47 (−2.62 – 2.32)
35–39 years	935 (791 – 1,090)	0.27 (0.22 – 0.31)	609 (503 – 737)	0.11 (0.09 – 0.13)	−2.72 (−2.83 – 2.62)
40–44 years	1,102 (939 – 1,295)	0.38 (0.33 – 0.45)	748 (611 – 896)	0.15 (0.12 – 0.18)	−3.16 (−3.28 – 3.04)
45–49 years	1,320 (1,099 – 1,600)	0.57 (0.47 – 0.69)	862 (701 – 1,042)	0.18 (0.15 – 0.22)	−3.7 (−3.77 – 3.64)
50–54 years	1,785 (1,489 – 2,088)	0.84 (0.7 – 0.98)	1,090 (893 – 1,292)	0.24 (0.2 – 0.29)	−4.06 (−4.17 – 3.96)
55–59 years	2,068 (1,788 – 2,394)	1.12 (0.97 – 1.29)	1,257 (1,045 – 1,508)	0.32 (0.26 – 0.38)	−4.07 (−4.23 – 3.9)
60–64 years	2,205 (1,915 – 2,552)	1.37 (1.19 – 1.59)	1,327 (1,119 – 1,584)	0.41 (0.35 – 0.49)	−4.04 (−4.2 – 3.88)
65–69 years	1,800 (1,562 – 2,075)	1.46 (1.26 – 1.68)	1,096 (939 – 1,260)	0.4 (0.34 – 0.46)	−4.08 (−4.24 – 3.92)
70–74 years	1,984 (1,720 – 2,301)	2.34 (2.03 – 2.72)	1,166 (1,018 – 1,336)	0.57 (0.49 – 0.65)	−4.17 (−4.29 – 4.05)
75–79 years	1,377 (1,185 – 1,574)	2.24 (1.92 – 2.56)	927 (809 – 1,067)	0.7 (0.61 – 0.81)	−3.99 (−4.11 – 3.86)
80–84 years	651 (559 – 745)	1.84 (1.58 – 2.1)	567 (486 – 650)	0.65 (0.56 – 0.74)	−3.51 (−3.75 – 3.26)
85–89 years	275 (228 – 320)	1.82 (1.51 – 2.12)	296 (253 – 348)	0.65 (0.55 – 0.76)	−3.15 (−3.41 – 2.89)
90–94 years	65 (50 – 79)	1.51 (1.16 – 1.84)	96 (77 – 116)	0.54 (0.43 – 0.65)	−2.91 (−3.11 – 2.72)
95+ years	12 (9 – 16)	1.22 (0.86 – 1.6)	25 (17 – 31)	0.46 (0.32 – 0.58)	−2.63 (−2.79 – 2.46)
**Sex**					
Female	9,159 (8,105 – 10,270)	0.4 (0.35 – 0.45)	5,873 (5,149 – 6,689)	0.14 (0.12 – 0.15)	−3.47 (−3.53 – 3.4)
Male	11,134 (9,844 – 12,507)	0.53 (0.47 – 0.59)	6,984 (6,104 – 7,940)	0.17 (0.15 – 0.2)	−3.61 (−3.72 – 3.51)
**SDI region**					
High-middle SDI	329 (286 – 378)	0.03 (0.03 – 0.04)	193 (157 – 238)	0.01 (0.01 – 0.01)	−3.93 (−4.1 – 3.76)
High SDI	36 (26 – 48)	0 (0 – 0)	42 (31 – 56)	0 (0 – 0)	−1.35 (−1.46 – 1.24)
Low-middle SDI	3,714 (3,353 – 4,086)	0.54 (0.5 – 0.6)	2,896 (2,521 – 3,294)	0.19 (0.16 – 0.21)	−3.1 (−3.34 – 2.87)
Low SDI	12,739 (11,047 – 14,622)	4.55 (3.98 – 5.15)	8,430 (7,265 – 9,681)	1.37 (1.2 – 1.56)	−3.94 (−4.08 – 3.81)
Middle SDI	3,467 (3,092 – 3,847)	0.32 (0.29 – 0.36)	1,291 (1,177 – 1,417)	0.05 (0.05 – 0.05)	−6.06 (−6.17 – 5.95)
**GBD region**					
*Advanced health system*	144 (118 – 170)	0.01 (0.01 – 0.01)	47 (36 – 60)	0 (0 – 0)	−4.19 (−4.51 – 3.87)
Africa	16,356 (14,378 – 18,448)	4.71 (4.18 – 5.25)	11,222 (9,749 – 12,757)	1.44 (1.27 – 1.63)	−3.74 (−3.9 – 3.58)
African Region	15,069 (13,194 – 17,097)	5.46 (4.81 – 6.12)	10,359 (8,974 – 11,846)	1.69 (1.49 – 1.91)	−3.76 (−3.94 – 3.57)
America	836 (777 – 897)	0.13 (0.12 – 0.14)	602 (547 – 654)	0.05 (0.04 – 0.05)	−3.17 (−3.28 – 3.06)
Asia	2,986 (2,608 – 3,384)	0.14 (0.12 – 0.16)	1,006 (885 – 1,136)	0.02 (0.02 – 0.02)	−6.48 (−6.72 – 6.25)
*Basic health system*	4,704 (4,284 – 5,114)	0.3 (0.28 – 0.33)	1,957 (1,779 – 2,143)	0.05 (0.05 – 0.06)	−5.44 (−5.52 – 5.35)
Caribbean	38 (29 – 49)	0.14 (0.1 – 0.17)	37 (27 – 48)	0.07 (0.05 – 0.09)	−1.47 (−1.91 – 1.03)
Central Africa	2,341 (1,909 – 2,808)	6.85 (5.65 – 8.23)	2,441 (1,948 – 2,966)	3.02 (2.48 – 3.66)	−2.57 (−2.89 – 2.26)
Central Latin America	42 (33 – 52)	0.04 (0.03 – 0.06)	50 (35 – 69)	0.02 (0.01 – 0.03)	−2.8 (−3.18 – 2.42)
Central Sub-Saharan Africa	2,048 (1,623 – 2,514)	7.5 (6.03 – 9.4)	1,984 (1,528 – 2,477)	3.02 (2.36 – 3.72)	−2.85 (−3.16 – 2.53)
Commonwealth High Income	0 (0 – 0)	0 (0 – 0)	0 (0 – 0)	0 (0 – 0)	−2.56 (−2.84 – 2.28)
Commonwealth Low Income	2,524 (2,162 – 2,915)	2.27 (1.96 – 2.6)	2,123 (1,770 – 2,609)	0.78 (0.65 – 0.96)	−3.46 (−3.79 – 3.14)
Commonwealth Middle Income	3,210 (2,722 – 3,691)	0.45 (0.38 – 0.52)	2,419 (1,966 – 2,885)	0.14 (0.12 – 0.17)	−3.78 (−4.08 – 3.47)
East Asia	2,523 (2,149 – 2,929)	0.29 (0.25 – 0.34)	527 (423 – 639)	0.03 (0.02 – 0.03)	−8.16 (−8.44 – 7.88)
East Asia & Pacific – WB	2,706 (2,334 – 3,111)	0.2 (0.17 – 0.23)	790 (674 – 917)	0.03 (0.02 – 0.03)	−6.84 (−7.1 – 6.58)
Eastern Africa	6,953 (5,965 – 8,221)	7.83 (6.73 – 9.21)	3,347 (2,978 – 3,810)	1.72 (1.53 – 1.95)	−5.04 (−5.17 – 4.92)
Eastern Mediterranean Region	1,567 (1,374 – 1,806)	0.77 (0.67 – 0.88)	1,079 (872 – 1,338)	0.21 (0.18 – 0.26)	−3.76 (−3.85 – 3.67)
Eastern Sub – Saharan Africa	7,921 (6,797 – 9,318)	8.3 (7.17 – 9.66)	4,251 (3,742 – 4,865)	2.05 (1.82 – 2.33)	−4.66 (−4.8 – 4.52)
Europe	108 (85 – 133)	0.01 (0.01 – 0.01)	21 (14 – 31)	0 (0 – 0)	−5.01 (−5.67 – 4.34)
Europe & Central Asia – WB	108 (85 – 133)	0.01 (0.01 – 0.01)	21 (14 – 31)	0 (0 – 0)	−5.02 (−5.69 – 4.35)
European Region	108 (85 – 133)	0.01 (0.01 – 0.01)	21 (14 – 31)	0 (0 – 0)	−5.03 (−5.7 – 4.36)
Latin America & Caribbean – WB	836 (777 – 897)	0.29 (0.26 – 0.31)	602 (547 – 654)	0.09 (0.08 – 0.09)	−3.72 (−3.84 – 3.6)
*Limited health system*	11,268 (9,783 – 12,857)	1.18 (1.04 – 1.33)	6,469 (5,679 – 7,328)	0.29 (0.26 – 0.33)	−4.59 (−4.76 – 4.42)
Middle East & North Africa – WB	1,180 (1,031 – 1,375)	0.89 (0.76 – 1.04)	579 (470 – 700)	0.17 (0.14 – 0.2)	−4.88 (−5.01 – 4.76)
*Minimal health system*	4,170 (3,564 – 4,842)	5.64 (4.85 – 6.55)	4,379 (3,580 – 5,173)	2.82 (2.35 – 3.33)	−2.05 (−2.22 – 1.89)
North Africa and Middle East	1,441 (1,275 – 1,640)	0.79 (0.69 – 0.9)	674 (550 – 813)	0.15 (0.12 – 0.18)	−4.92 (−5.05 – 4.8)
Northern Africa	930 (788 – 1,097)	1.42 (1.19 – 1.69)	375 (298 – 468)	0.25 (0.2 – 0.31)	−4.75 (−4.98 – 4.51)
Region of the Americas	836 (777 – 897)	0.13 (0.12 – 0.14)	602 (547 – 654)	0.05 (0.04 – 0.05)	−3.17 (−3.28 – 3.06)
South-East Asia Region	10 (6 – 15)	0 (0 – 0)	13 (7 – 21)	0 (0 – 0)	−1.94 (−2.25 – 1.63)
Southeast Asia	190 (169 – 211)	0.06 (0.06 – 0.07)	267 (230 – 307)	0.04 (0.03 – 0.05)	−1.08 (−1.26 – 0.89)
Southern Africa	1,428 (1,214 – 1,650)	2.56 (2.2 – 2.97)	1,402 (1,145 – 1,677)	1.21 (1.01 – 1.43)	−2.26 (−2.6 – 1.91)
Southern Sub-Saharan Africa	226 (197 – 259)	0.69 (0.6 – 0.79)	371 (305 – 445)	0.56 (0.47 – 0.68)	−0.39 (−1.12 – 0.35)
Sub-Saharan Africa – WB	15,456 (13,547 – 17,514)	5.6 (4.95 – 6.28)	10,860 (9,430 – 12,379)	1.79 (1.58 – 2.03)	−3.64 (−3.82 – 3.47)
Tropical Latin America	757 (699 – 817)	0.76 (0.7 – 0.83)	517 (467 – 564)	0.2 (0.18 – 0.22)	−4.07 (−4.19 – 3.95)
Western Africa	4,703 (4,046 – 5,399)	4.88 (4.28 – 5.61)	3,656 (3,026 – 4,300)	1.71 (1.46 – 1.98)	−3.22 (−3.43 – 3.02)
Western Pacific Region	2,696 (2,321 – 3,099)	0.23 (0.2 – 0.26)	777 (661 – 904)	0.03 (0.02 – 0.03)	−6.88 (−7.14 – 6.62)
Western Sub-Saharan Africa	5,108 (4,393 – 5,833)	4.8 (4.18 – 5.48)	4,180 (3,445 – 4,930)	1.74 (1.49 – 2.02)	−3.11 (−3.34 – 2.89)
World Bank High Income	36 (26 – 49)	0 (0 – 0)	26 (18 – 36)	0 (0 – 0)	−2.38 (−2.5 – 2.25)
World Bank Low Income	10,383 (8,913 – 12,074)	5.65 (4.91 – 6.53)	6,676 (5,678 – 7,705)	1.68 (1.45 – 1.93)	−4.03 (−4.15 – 3.91)
World Bank Lower Middle Income	6,118 (5,443 – 6,761)	0.51 (0.46 – 0.57)	4,770 (4,137 – 5,448)	0.17 (0.15 – 0.2)	−3.2 (−3.43 – 2.98)
World Bank Upper Middle Income	3,748 (3,346 – 4,153)	0.24 (0.22 – 0.27)	1,380 (1,247 – 1,513)	0.04 (0.04 – 0.05)	−5.78 (−5.91 – 5.64)
**Country**					
Algeria	26 (18 – 36)	0.21 (0.14 – 0.29)	25 (18 – 35)	0.08 (0.05 – 0.11)	−3.03 (−3.06 – 2.99)
Angola	366 (271 – 494)	7.3 (5.43 – 9.88)	263 (187 – 351)	1.83 (1.34 – 2.38)	−4.54 (−4.82 – 4.26)
Antigua and Barbuda	0 (0 – 0)	0.34 (0.16 – 0.57)	0 (0 – 0)	0.14 (0.06 – 0.24)	−2.96 (−3.24 – 2.67)
Benin	170 (133 – 216)	6.69 (5.18 – 8.55)	155 (115 – 199)	2.39 (1.8 – 3.08)	−3.19 (−3.38 – 3)
Botswana	22 (13 – 32)	3.21 (1.91 – 4.8)	16 (10 – 23)	0.96 (0.58 – 1.41)	−4.06 (−4.71 – 3.41)
Brazil	757 (699 – 817)	0.78 (0.72 – 0.85)	517 (467 – 564)	0.21 (0.19 – 0.23)	−4.07 (−4.19 – 3.96)
Burkina Faso	310 (241 – 385)	5.97 (4.73 – 7.5)	314 (238 – 403)	2.76 (2.13 – 3.55)	−2.27 (−2.51 – 2.04)
Burundi	286 (213 – 375)	9.76 (7.2 – 12.78)	211 (161 – 274)	3.39 (2.56 – 4.51)	−3.98 (−4.25 – 3.72)
Cambodia	17 (11 – 25)	0.32 (0.2 – 0.47)	20 (12 – 31)	0.16 (0.1 – 0.25)	−2.21 (−2.29 – 2.13)
Cameroon	268 (207 – 329)	4.91 (3.82 – 6.04)	324 (233 – 450)	2.08 (1.46 – 2.85)	−2.55 (−3.06 – 2.04)
Central African Republic	215 (163 – 274)	15.5 (11.98 – 19.59)	277 (194 – 383)	9.91 (6.98 – 13.26)	−1.48 (−1.64 – 1.31)
Chad	106 (81 – 135)	3.23 (2.41 – 4.21)	185 (138 – 243)	2.41 (1.79 – 3.14)	−1.08 (−1.51 – 0.64)
China	2,523 (2,149 – 2,929)	0.31 (0.26 – 0.35)	527 (423 – 639)	0.03 (0.02 – 0.03)	−8.16 (−8.44 – 7.88)
Congo	102 (77 – 134)	8.21 (6.22 – 10.73)	70 (50 – 96)	2.21 (1.57 – 2.95)	−4.62 (−4.94 – 4.3)
Saint Helena	266 (204 – 346)	4.9 (3.77 – 6.25)	244 (176 – 336)	1.79 (1.31 – 2.41)	−3 (−3.5 – 2.5)
Democratic Republic of the Congo	1,324 (994 – 1,738)	7.05 (5.36 – 9.3)	1,356 (987 – 1,788)	3.14 (2.32 – 4.16)	−2.43 (−2.8 – 2.06)
Djibouti	1 (0 – 1)	0.29 (0.13 – 0.5)	2 (1 – 3)	0.22 (0.09 – 0.38)	−1.08 (−1.35 – 0.81)
Dominican Republic	34 (25 – 44)	0.8 (0.58 – 1.04)	33 (23 – 44)	0.32 (0.23 – 0.42)	−2.15 (−2.65 – 1.65)
Egypt	783 (646 – 959)	2.9 (2.33 – 3.56)	253 (186 – 338)	0.44 (0.33 – 0.59)	−4.93 (−5.26 – 4.6)
Equatorial Guinea	8 (5 – 13)	3.62 (1.94 – 5.75)	3 (2 – 5)	0.49 (0.22 – 0.85)	−7.06 (−7.58 – 6.54)
Eritrea	120 (92 – 158)	7.53 (5.64 – 9.83)	109 (75 – 151)	3.16 (2.26 – 4.3)	−2.64 (−2.76 – 2.52)
Eswatini	6 (3 – 9)	1.56 (0.79 – 2.52)	9 (5 – 14)	1.21 (0.63 – 1.93)	−0.18 (−1.18 – 0.84)
Ethiopia	4,153 (3,423 – 5,247)	16.8 (13.97 – 21.12)	878 (721 – 1,055)	1.74 (1.42 – 2.09)	−7.73 (−7.96 – 7.5)
Gabon	33 (23 – 45)	5.31 (3.73 – 7.36)	15 (9 – 23)	1.34 (0.8 – 2.06)	−4.44 (−4.77 – 4.1)
Gambia	11 (6 – 16)	2.41 (1.25 – 3.85)	16 (10 – 24)	1.38 (0.81 – 2.12)	−2 (−2.23 – 1.77)
Ghana	379 (291 – 475)	4.84 (3.67 – 6.12)	343 (249 – 448)	1.83 (1.35 – 2.39)	−2.5 (−2.77 – 2.23)
Guinea	217 (169 – 273)	5.43 (4.22 – 6.87)	182 (133 – 246)	2.68 (1.93 – 3.65)	−1.91 (−2.14 – 1.67)
Guinea-Bissau	47 (33 – 64)	9.21 (6.56 – 12.85)	35 (23 – 50)	3.75 (2.48 – 5.3)	−2.77 (−2.96 – 2.58)
Indonesia	10 (6 – 15)	0.01 (0 – 0.01)	13 (7 – 21)	0 (0 – 0.01)	−1.61 (−1.92 – 1.3)
Iran (Islamic Republic of)	4 (2 – 6)	0.02 (0.01 – 0.03)	3 (1 – 5)	0 (0 – 0.01)	−4.33 (−4.48 – 4.19)
Iraq	69 (53 – 89)	0.75 (0.57 – 0.99)	44 (31 – 60)	0.19 (0.13 – 0.25)	−5.32 (−5.68 – 4.95)
Jordan	3 (1 – 4)	0.18 (0.08 – 0.29)	3 (2 – 5)	0.05 (0.02 – 0.07)	−5.11 (−5.5 – 4.72)
Kenya	240 (200 – 277)	2.45 (2.03 – 2.88)	353 (284 – 432)	1.4 (1.12 – 1.73)	−0.84 (−1.47 – 0.21)
Lao People’s Democratic Republic	18 (12 – 26)	0.76 (0.49 – 1.13)	12 (7 – 19)	0.25 (0.14 – 0.38)	−3.79 (−3.95 – 3.63)
Lebanon	1 (0 – 1)	0.04 (0.02 – 0.07)	2 (1 – 3)	0.03 (0.01 – 0.05)	−0.99 (−1.13 – 0.85)
Liberia	96 (72 – 122)	6.91 (5.27 – 8.97)	52 (34 – 73)	1.95 (1.27 – 2.81)	−4.37 (−4.61 – 4.13)
Libya	10 (6 – 15)	0.49 (0.26 – 0.75)	11 (6 – 17)	0.2 (0.12 – 0.33)	−2.8 (−3.11 – 2.48)
Madagascar	349 (287 – 419)	5.3 (4.3 – 6.42)	297 (220 – 400)	2.09 (1.55 – 2.76)	−2.81 (−2.92 – 2.7)
Malawi	266 (208 – 328)	5.26 (4.15 – 6.53)	188 (144 – 245)	2.03 (1.56 – 2.7)	−3.27 (−3.69 – 2.86)
Mali	377 (301 – 468)	7.52 (6.05 – 9.25)	314 (243 – 413)	2.9 (2.2 – 3.87)	−2.64 (−2.86 – 2.43)
Mauritania	31 (21 – 43)	2.76 (1.83 – 3.9)	14 (9 – 22)	0.61 (0.35 – 0.95)	−4.73 (−4.84 – 4.62)
Mauritius	6 (3 – 10)	0.86 (0.39 – 1.33)	4 (2 – 6)	0.23 (0.13 – 0.36)	−3.96 (−4.31 – 3.61)
Morocco	70 (54 – 90)	0.45 (0.35 – 0.57)	63 (46 – 81)	0.19 (0.14 – 0.24)	−2.63 (−2.71 – 2.55)
Mozambique	336 (257 – 430)	4.49 (3.4 – 5.71)	401 (286 – 540)	2.8 (2.03 – 3.68)	−0.66 (−1 – 0.32)
Namibia	26 (18 – 35)	3.56 (2.5 – 4.81)	19 (11 – 29)	1.26 (0.77 – 1.97)	−3.59 (−4.21 – 2.97)
Niger	209 (162 – 264)	5.19 (3.95 – 6.61)	214 (159 – 280)	2.03 (1.48 – 2.72)	−3.15 (−3.37 – 2.93)
Nigeria	2,164 (1,765 – 2,591)	4.25 (3.47 – 5.1)	1,411 (1,076 – 1,808)	1.25 (0.99 – 1.57)	−3.97 (−4.28 – 3.65)
Oman	3 (1 – 5)	0.39 (0.18 – 0.72)	1 (1 – 3)	0.08 (0.03 – 0.14)	−4.55 (−4.79 – 4.3)
Philippines	137 (123 – 153)	0.39 (0.35 – 0.44)	217 (184 – 256)	0.26 (0.22 – 0.3)	−0.84 (−1.05 – 0.63)
Rwanda	389 (301 – 486)	10.57 (8.01 – 13.18)	116 (85 – 156)	1.63 (1.17 – 2.23)	−7.43 (−8.03 – 6.83)
Saint Lucia	0 (0 – 0)	0.01 (0.01 – 0.02)	0 (0 – 0)	0.01 (0.01 – 0.02)	−0.07 (−0.22 – 0.07)
Sao Tome and Principe	0 (0 – 0)	0.05 (0.02 – 0.08)	0 (0 – 0)	0.05 (0.02 – 0.09)	0.2 (0.07 – 0.33)
Saudi Arabia	33 (23 – 45)	0.48 (0.32 – 0.67)	24 (17 – 35)	0.11 (0.07 – 0.16)	−4.96 (−5.11 – 4.8)
Senegal	279 (220 – 345)	6.79 (5.32 – 8.55)	189 (140 – 256)	2.18 (1.62 – 2.97)	−3.16 (−3.34 – 2.99)
Sierra Leone	102 (76 – 130)	4.02 (2.95 – 5.19)	88 (62 – 122)	1.87 (1.33 – 2.56)	−1.97 (−2.34 – 1.61)
Somalia	260 (189 – 351)	7.57 (5.68 – 9.93)	451 (319 – 615)	5.27 (3.74 – 7.05)	−1.17 (−1.3 – 1.03)
South Africa	105 (89 – 123)	0.4 (0.34 – 0.46)	115 (98 – 134)	0.23 (0.2 – 0.27)	−2.19 (−3.15 – 1.22)
South Sudan	90 (66 – 119)	2.84 (2 – 3.85)	85 (59 – 116)	1.71 (1.17 – 2.34)	−1.83 (−2.02 – 1.64)
Sudan	153 (120 – 202)	1.36 (1.05 – 1.76)	74 (51 – 106)	0.33 (0.24 – 0.47)	−4.49 (−4.58 – 4.4)
Suriname	3 (1 – 4)	1.02 (0.51 – 1.6)	3 (1 – 4)	0.42 (0.21 – 0.69)	−2.86 (−3.04 – 2.69)
Syrian Arab Republic	22 (14 – 31)	0.38 (0.24 – 0.56)	14 (8 – 21)	0.11 (0.07 – 0.18)	−4.17 (−4.55 – 3.78)
Togo	76 (59 – 95)	4.72 (3.58 – 6.04)	100 (70 – 140)	2.23 (1.56 – 3.12)	−2.23 (−2.63 – 1.83)
Tunisia	11 (7 – 16)	0.21 (0.12 – 0.3)	9 (5 – 14)	0.07 (0.04 – 0.11)	−3.59 (−3.67 – 3.5)
Turkey	108 (85 – 133)	0.3 (0.23 – 0.37)	21 (14 – 31)	0.02 (0.02 – 0.04)	−6.36 (−7.01 – 5.7)
Uganda	487 (377 – 638)	6.25 (4.74 – 8.16)	413 (316 – 537)	2.15 (1.62 – 2.84)	−4.12 (−4.75 – 3.48)
United Republic of Tanzania	704 (558 – 875)	5.07 (3.97 – 6.4)	564 (423 – 747)	1.83 (1.4 – 2.45)	−3.09 (−3.25 – 2.92)
Venezuela (Bolivarian Republic of)	42 (33 – 52)	0.38 (0.29 – 0.47)	50 (35 – 69)	0.17 (0.12 – 0.24)	−2.87 (−3.29 – 2.46)
Yemen	146 (104 – 202)	2.61 (1.89 – 3.53)	125 (85 – 179)	0.81 (0.57 – 1.16)	−4.08 (−4.31 – 3.85)
Zambia	235 (185 – 295)	5.98 (4.67 – 7.48)	179 (126 – 247)	1.99 (1.4 – 2.73)	−4.12 (−4.72 – 3.51)
Zimbabwe	68 (51 – 88)	1.46 (1.08 – 1.94)	213 (158 – 279)	2.45 (1.83 – 3.22)	3.27 (2.37 – 4.17)

UI: uncertainty intervals; CI: confidence interval; SDI: socio-demographic index; EAPC: estimated annual percent change.

**Table 2:** Number of DALYs cases and age-standardized DALYs rate (ASDR) attributable to schistosomiasis in 1990 and 2021, and trends from 1990 to 2021 globally.

Characteristics	Number of DALYs cases (95% UI) in 1990	The age – standardized DALYs rate/100,000 (95% UI) in 1990	Number of DALYs cases (95% UI) in 2021	The age – standardized DALYs rate/100,000 (95% UI) in 2021	EAPC (95% CI)
Global	1,946,769 (1,366,510 – 3,027,337)	37.35 (26.78 – 57.05)	1,746,333 (1,038,122 – 2,984,204)	21.9 (12.94 – 37.28)	−1.56 (−1.78 – 1.34)
**Age**					
<5 years	72,666 (53,552 – 98,427)	11.72 (8.64 – 15.88)	31,379 (20,041 – 42,823)	4.77 (3.05 – 6.51)	−2.46 (−2.74 – 2.19)
5–9 years	151,354 (97,657 – 246,149)	25.94 (16.74 – 42.18)	64,173 (36,514 – 115,281)	9.34 (5.31 – 16.78)	−1.84 (−2.77 – 0.9)
10–14 years	209,821 (119,401 – 367,671)	39.17 (22.29 – 68.64)	154,410 (69,946 – 316,401)	23.16 (10.49 – 47.46)	−0.78 (−1.34 – 0.22)
15–19 years	212,660 (118,792 – 374,059)	40.94 (22.87 – 72.01)	221,230 (104,047 – 417,994)	35.45 (16.67 – 66.99)	−0.37 (−0.57 – 0.17)
20–24 years	201,323 (117,632 – 340,817)	40.91 (23.9 – 69.26)	216,793 (104,294 – 400,763)	36.3 (17.46 – 67.11)	−0.58 (−0.82 – 0.34)
25–29 years	179,355 (106,042 – 320,081)	40.52 (23.96 – 72.32)	194,408 (98,488 – 379,521)	33.04 (16.74 – 64.51)	−0.77 (−1.04 – 0.51)
30–34 years	151,650 (93,888 – 268,601)	39.35 (24.36 – 69.69)	170,529 (88,105 – 336,500)	28.21 (14.58 – 55.67)	−1.01 (−1.23 – 0.8)
35–39 years	135,944 (90,390 – 223,597)	38.59 (25.66 – 63.48)	142,839 (78,157 – 257,221)	25.47 (13.94 – 45.86)	−1.3 (−1.43 – 1.16)
40–44 years	114,725 (80,031 – 172,998)	40.05 (27.94 – 60.39)	120,235 (72,695 – 215,901)	24.03 (14.53 – 43.16)	−1.77 (−1.87 – 1.67)
45–49 years	102,290 (77,492 – 149,077)	44.05 (33.37 – 64.2)	100,729 (66,816 – 160,435)	21.27 (14.11 – 33.88)	−2.42 (−2.48 – 2.37)
50–54 years	101,443 (80,455 – 135,529)	47.72 (37.85 – 63.76)	88,875 (63,432 – 132,610)	19.98 (14.26 – 29.81)	−3 (−3.11 – 2.89)
55–59 years	92,691 (74,700 – 117,059)	50.05 (40.33 – 63.21)	74,813 (55,791 – 106,016)	18.91 (14.1 – 26.79)	−3.25 (−3.41 – 3.1)
60–64 years	80,028 (67,801 – 96,213)	49.83 (42.21 – 59.91)	58,788 (45,291 – 78,840)	18.37 (14.15 – 24.63)	−3.42 (−3.58 – 3.26)
65–69 years	54,909 (46,226 – 66,156)	44.42 (37.4 – 53.52)	40,990 (32,303 – 54,490)	14.86 (11.71 – 19.75)	−3.48 (−3.64 – 3.32)
70–74 years	46,484 (39,731 – 54,522)	54.91 (46.93 – 64.4)	31,784 (26,124 – 40,635)	15.44 (12.69 – 19.74)	−3.74 (−3.84 – 3.63)
75–79 years	25,612 (21,984 – 30,109)	41.61 (35.71 – 48.91)	19,437 (16,241 – 24,884)	14.74 (12.31 – 18.87)	−3.64 (−3.76 – 3.52)
80–84 years	9,694 (8,181 – 11,435)	27.4 (23.12 – 32.32)	9,470 (7,662 – 12,401)	10.81 (8.75 – 14.16)	−3.18 (−3.42 – 2.95)
85–89 years	3,287 (2,756 – 3,989)	21.75 (18.24 – 26.4)	3,976 (3,234 – 5,164)	8.7 (7.07 – 11.29)	−2.83 (−3.08 – 2.58)
90–94 years	702 (550 – 874)	16.39 (12.84 – 20.4)	1,182 (926 – 1,551)	6.61 (5.18 – 8.67)	−2.57 (−2.76 – 2.38)
95+ years	129 (92 – 169)	12.64 (9.06 – 16.57)	295 (203 – 407)	5.41 (3.73 – 7.47)	−2.32 (−2.46 – 2.19)
**Sex**					
Female	927,632 (648,016 – 1,456,133)	35.47 (25.28 – 54.71)	855,353 (512,420 – 1,439,690)	21.65 (12.89 – 36.59)	−1.44 (−1.65 – 1.23)
Male	1,019,138 (709,474 – 1,588,963)	39.36 (28.24 – 60.59)	890,980 (531,972 – 1,563,697)	22.17 (13.17 – 38.75)	−1.68 (−1.91 – 1.45)
**SDI region**					
High-middle SDI	28,295 (18,194 – 47,845)	2.6 (1.7 – 4.36)	16,709 (9,715 – 31,289)	1.2 (0.66 – 2.34)	−2.63 (−2.91 – 2.34)
High SDI	3,155 (1,888 – 5,197)	0.36 (0.21 – 0.6)	5,319 (2,691 – 10,549)	0.49 (0.23 – 0.99)	0.97 (0.79 – 1.15)
Low-middle SDI	502,465 (319,420 – 845,647)	48.15 (32.24 – 77.94)	524,056 (292,918 – 929,383)	26.81 (15.39 – 48.05)	−1.98 (−2.21 – 1.75)
Low SDI	1,071,134 (796,732 – 1,576,538)	263.14 (203.5 – 365.51)	891,679 (582,022 – 1,426,191)	94.48 (65.77 – 146.99)	−3.1 (−3.4 – 2.81)
Middle SDI	340,834 (216,665 – 576,954)	21.98 (14.96 – 35.37)	307,641 (155,918 – 611,479)	12.1 (6.1 – 24.15)	−1.96 (−2.06 – 1.86)
**GBD region**					
*Advanced Health System*	9,458 (6,675 – 14,539)	0.69 (0.48 – 1.07)	6,154 (3,042 – 12,136)	0.41 (0.19 – 0.83)	−2.23 (−2.76 – 1.7)
Africa	1,629,753 (1,146,955 – 2,530,831)	313.6 (230.85 – 461.15)	1,535,380 (921,967 – 2,581,477)	126.43 (80.66 – 211.13)	−2.82 (−3.08 – 2.55)
African Region	1,424,185 (1,016,194 – 2,171,924)	346.42 (258.41 – 502.1)	1,466,548 (871,943 – 2,483,660)	147.6 (94.01 – 247.37)	−2.62 (−2.87 – 2.37)
America	70,591 (48,519 – 114,570)	10.09 (7.12 – 16.08)	71,901 (41,722 – 129,277)	6.54 (3.7 – 12.02)	−1.26 (−1.42 – 1.1)
Asia	239,496 (161,777 – 382,551)	8.47 (6.01 – 13.01)	137,607 (75,078 – 262,677)	2.79 (1.51 – 5.41)	−3.63 (−3.71 – 3.55)
*Basic Health System*	505,802 (322,288 – 825,444)	23.37 (15.73 – 36.97)	284,297 (158,202 – 538,990)	8.55 (4.67 – 16.46)	−3.39 (−3.54 – 3.24)
Caribbean	7,427 (3,984 – 14,656)	21 (11.63 – 40.35)	9,921 (4,739 – 21,092)	20.28 (9.6 – 43.34)	0.01 (−0.09 – 0.11)
Central Africa	200,041 (144,351 – 304,303)	390.68 (294.74 – 561.01)	199,854 (141,311 – 304,173)	159.78 (117.77 – 228.26)	−2.31 (−2.79 – 1.83)
Central Latin America	5,313 (3,165 – 9,606)	3.65 (2.33 – 6.25)	6,405 (3,429 – 12,421)	2.43 (1.31 – 4.7)	−0.88 (−1.01 – 0.74)
Central Sub-Saharan Africa	167,543 (120,049 – 250,650)	408.03 (311.35 – 570.13)	165,861 (113,450 – 255,590)	161.89 (116.49 – 234.2)	−2.34 (−2.75 – 1.93)
Commonwealth High Income	36 (11 – 88)	0.03 (0.01 – 0.08)	48 (12 – 123)	0.03 (0.01 – 0.09)	0.28 (0.11 – 0.46)
Commonwealth Low Income	292,511 (200,155 – 471,454)	167.29 (119.66 – 255.82)	255,453 (156,933 – 433,423)	69.26 (44.83 – 113.55)	−2.62 (−3.01 – 2.23)
Commonwealth Middle Income	384,446 (245,225 – 622,479)	36.44 (24.4 – 57.3)	614,718 (331,133 – 1,097,381)	28.04 (15.44 – 50.11)	−0.99 (−1.15 – 0.83)
East Asia	190,712 (130,309 – 300,095)	16.65 (11.69 – 25.27)	86,749 (43,666 – 167,202)	5.35 (2.55 – 10.63)	−3.76 (−4.03 – 3.49)
East Asia & Pacific – WB	207,330 (141,156 – 329,827)	11.76 (8.3 – 18.01)	100,585 (54,854 – 187,128)	3.8 (1.97 – 7.24)	−3.58 (−3.75 – 3.4)
Eastern Africa	574,567 (425,826 – 842,673)	431.42 (332.76 – 592.9)	488,727 (287,736 – 862,149)	149.21 (94.85 – 253.64)	−3.45 (−3.6 – 3.3)
Eastern Mediterranean Region	237,735 (150,218 – 395,815)	72.99 (48.64 – 116.9)	105,854 (68,548 – 176,015)	15.25 (10.4 – 24.67)	−5.31 (−5.83 – 4.79)
Eastern Sub-Saharan Africa	645,887 (483,082 – 949,198)	447.64 (348.75 – 612.35)	552,263 (332,713 – 948,844)	157.58 (103.56 – 261.73)	−3.37 (−3.55 – 3.18)
Europe	6,042 (4,342 – 8,744)	0.72 (0.51 – 1.06)	516 (350 – 751)	0.04 (0.03 – 0.06)	−8.54 (−9.46 – 7.61)
Europe & Central Asia – WB	6,042 (4,342 – 8,744)	0.68 (0.48 – 1)	516 (350 – 751)	0.04 (0.03 – 0.06)	−8.56 (−9.48 – 7.63)
European Region	6,042 (4,342 – 8,744)	0.67 (0.48 – 0.99)	516 (350 – 751)	0.04 (0.03 – 0.06)	−8.57 (−9.49 – 7.64)
Latin America & Caribbean – WB	70,591 (48,519 – 114,570)	18.27 (13.27 – 28.05)	71,901 (41,722 – 129,277)	10.25 (5.93 – 18.56)	−1.71 (−1.9 – 1.53)
*Limited Health System*	1,080,126 (765,670 – 1,657,604)	78.65 (58.02 – 115.45)	1,145,322 (649,660 – 2,009,003)	39.28 (22.87 – 68.5)	−2.27 (−2.46 – 2.08)
Middle East & North Africa – WB	209,131 (126,812 – 360,551)	92.28 (59.41 – 152.27)	78,525 (45,439 – 141,970)	16.74 (10.02 – 29.66)	−6 (−6.6 – 5.4)
*Minimal Health System*	350,497 (259,024 – 509,811)	323.63 (249.26 – 452.77)	309,631 (231,063 – 444,801)	130.45 (101.34 – 173.15)	−2.38 (−2.81 – 1.95)
North Africa and Middle East	227,874 (141,337 – 386,137)	75.8 (49.93 – 122.77)	84,796 (49,989 – 151,197)	13.86 (8.46 – 24.21)	−5.83 (−6.4 – 5.26)
Northern Africa	179,286 (107,283 – 310,497)	162.85 (103.67 – 271.18)	42,942 (27,019 – 74,087)	21.51 (13.88 – 36.08)	−6.89 (−7.69 – 6.07)
Region of the Americas	70,591 (48,519 – 114,570)	10.09 (7.12 – 16.08)	71,901 (41,722 – 129,277)	6.54 (3.7 – 12.02)	−1.26 (−1.42 – 1.1)
South-East Asia Region	572 (348 – 870)	0.05 (0.03 – 0.08)	517 (316 – 825)	0.02 (0.02 – 0.04)	−2.31 (−2.74 – 1.88)
Southeast Asia	20,400 (13,189 – 35,935)	4.89 (3.33 – 8.28)	17,805 (12,662 – 27,702)	2.44 (1.74 – 3.78)	−1.72 (−2.06 – 1.37)
Southern Africa	135,982 (94,748 – 208,216)	170.73 (126.34 – 250.61)	141,764 (93,413 – 227,693)	87.55 (60.11 – 137.13)	−1.75 (−2.11 – 1.4)
Southern Sub-Saharan Africa	43,710 (24,831 – 76,928)	89.86 (54.15 – 154.06)	55,643 (32,530 – 98,937)	68.76 (41.6 – 120.94)	−0.62 (−1.01 – 0.21)
Sub-Saharan Africa – WB	1,452,788 (1,039,102 – 2,209,350)	352.91 (263.31 – 509.52)	1,493,877 (895,646 – 2,513,410)	150.11 (96.68 – 249.61)	−2.62 (−2.87 – 2.36)
Tropical Latin America	58,102 (41,319 – 91,267)	43.43 (32.47 – 63.77)	55,911 (33,103 – 95,821)	22.64 (13.23 – 39.49)	−1.99 (−2.21 – 1.76)
Western Africa	539,878 (361,108 – 849,702)	369.56 (258.41 – 565.35)	662,093 (378,540 – 1,140,464)	177.69 (108.35 – 303.96)	−2.34 (−2.59 – 2.08)
Western Pacific Region	206,758 (140,569 – 328,889)	13.95 (9.83 – 21.44)	100,067 (54,506 – 186,624)	4.7 (2.41 – 9)	−3.45 (−3.64 – 3.27)
Western Sub-Saharan Africa	579,802 (389,643 – 915,839)	359.32 (253 – 548.34)	710,979 (413,883 – 1,212,784)	171.13 (106.54 – 289.25)	−2.34 (−2.63 – 2.05)
World Bank High Income	3,399 (2,025 – 5,658)	0.34 (0.2 – 0.58)	5,598 (2,548 – 11,527)	0.48 (0.21 – 1.02)	1.15 (0.94 – 1.35)
World Bank Low Income	835,064 (620,718 – 1,220,303)	316.72 (244.81 – 434.49)	665,974 (439,144 – 1,065,816)	111.33 (78.84 – 171.68)	−3.13 (−3.4 – 2.86)
World Bank Lower Middle Income	794,299 (520,260 – 1,297,696)	44.89 (30.65 – 71.59)	841,642 (476,906 – 1,465,729)	24.77 (14.36 – 42.55)	−1.96 (−2.22 – 1.7)
World Bank Upper Middle Income	326,810 (214,251 – 540,934)	16.48 (11.32 – 26.21)	239,949 (126,169 – 468,844)	8.7 (4.45 – 17.37)	−2.03 (−2.22 – 1.85)
**Country**					
Algeria	1,570 (1,016 – 2,338)	8.25 (5.64 – 11.61)	1,775 (1,097 – 3,060)	4.23 (2.71 – 7.11)	−2.13 (−2.19 – 2.07)
Angola	24,112 (17,458 – 34,296)	336.37 (247.22 – 462.38)	25,735 (16,330 – 42,866)	107.47 (73.18 – 165.35)	−3.36 (−3.61 – 3.11)
Antigua and Barbuda	36 (11 – 88)	58.7 (19.58 – 140.13)	48 (12 – 123)	49.54 (11.46 – 126.26)	−0.52 (−0.58 – 0.47)
Benin	19,381 (13,076 – 30,311)	485.52 (343.49 – 725.4)	19,505 (12,216 – 32,076)	178.54 (117.35 – 283.17)	−2.12 (−2.87 – 1.35)
Botswana	3,009 (1,678 – 5,253)	266.82 (160.26 – 435.51)	3,450 (1,822 – 6,600)	140.88 (76.79 – 263.21)	−1.61 (−1.84 – 1.38)
Brazil	58,102 (41,319 – 91,267)	44.52 (33.28 – 65.37)	55,911 (33,103 – 95,821)	23.33 (13.62 – 40.76)	−1.97 (−2.19 – 1.74)
Burkina Faso	32,563 (21,711 – 50,356)	400.43 (282.44 – 597.27)	16,769 (12,975 – 21,728)	106.14 (82.8 – 135.63)	−4.33 (−4.98 – 3.67)
Burundi	19,054 (13,757 – 27,805)	467.8 (344.36 – 643.83)	12,360 (9,123 – 16,688)	137.44 (104.03 – 180.18)	−3.94 (−4.3 – 3.58)
Cambodia	769 (486 – 1,150)	11.32 (7.02 – 16.28)	676 (417 – 1,015)	4.65 (2.85 – 7.03)	−3.01 (−3.15 – 2.86)
Cameroon	30,311 (20,580 – 48,066)	344.39 (243.17 – 527.72)	34,909 (22,876 – 57,009)	136.03 (95.45 – 211.74)	−2.78 (−3.54 – 2.01)
Central African Republic	11,263 (8,897 – 14,473)	619.39 (490.35 – 783.48)	15,513 (11,056 – 21,693)	401.55 (289.82 – 540.61)	−1.3 (−1.48 – 1.12)
Chad	7,243 (5,195 – 10,581)	161.92 (117.8 – 228.33)	12,456 (9,130 – 17,542)	104.13 (78.87 – 140.26)	−0.8 (−1.26 – 0.34)
China	190,712 (130,309 – 300,095)	17.26 (12.13 – 26.17)	86,749 (43,666 – 167,202)	5.55 (2.65 – 11.03)	−3.76 (−4.03 – 3.48)
Congo	7,219 (5,273 – 10,452)	405.59 (305.81 – 553.79)	6,591 (4,135 – 10,932)	140.53 (91.89 – 215.07)	−2.69 (−3.1 – 2.27)
Saint Helena	43,768 (27,858 – 74,135)	414.73 (279.16 – 666.02)	25,160 (16,573 – 39,767)	111.81 (78.12 – 168.8)	−3.67 (−4.27 – 3.07)
Democratic Republic of the Congo	121,393 (83,516 – 186,195)	416.21 (302.65 – 596.3)	115,995 (77,572 – 178,622)	170.49 (116.43 – 247.13)	−2.12 (−2.6 – 1.64)
Djibouti	28 (13 – 61)	10.04 (4.5 – 19.72)	70 (28 – 159)	7.18 (2.92 – 14.65)	−1.24 (−1.54 – 0.94)
Dominican Republic	6,742 (3,566 – 13,360)	101.82 (57.61 – 193.73)	9,072 (4,302 – 19,366)	79.34 (38.03 – 168.21)	−0.66 (−0.78 – 0.55)
Egypt	167,074 (98,156 – 290,203)	331.2 (208.15 – 553.1)	29,646 (19,463 – 49,033)	32.36 (21.85 – 50.77)	−7.83 (−8.8 – 6.85)
Equatorial Guinea	448 (292 – 645)	145.66 (92.3 – 216.91)	458 (239 – 867)	35.98 (20.65 – 61.67)	−4.98 (−5.44 – 4.51)
Eritrea	7,986 (6,081 – 10,823)	334.22 (259.41 – 432.69)	5,728 (4,052 – 7,833)	117.8 (85.92 – 158.58)	−2.79 (−3.13 – 2.45)
Eswatini	419 (244 – 652)	75.22 (46.72 – 114.03)	555 (330 – 877)	57.36 (34.35 – 90.73)	−0.18 (−1.02 – 0.66)
Ethiopia	254,174 (198,950 – 340,361)	720.29 (578.83 – 923.51)	192,008 (101,803 – 356,501)	206.47 (120.15 – 368.31)	−4.49 (−4.68 – 4.29)
Gabon	3,108 (1,990 – 5,004)	369.83 (247.59 – 575.06)	1,568 (910 – 2,716)	96.85 (59.7 – 162.11)	−3.01 (−3.57 – 2.44)
Gambia	1,095 (633 – 1,833)	147.29 (91.21 – 235.17)	1,403 (837 – 2,277)	74.95 (46.76 – 111.03)	−1.36 (−1.71 – 1)
Ghana	67,825 (40,673 – 114,671)	512.35 (328.26 – 831.5)	71,471 (37,938 – 129,143)	225.96 (129.06 – 400.77)	−2.38 (−2.83 – 1.92)
Guinea	18,684 (13,373 – 27,643)	349.43 (258.93 – 499.04)	13,098 (9,015 – 20,050)	130.49 (94.72 – 184.53)	−2.16 (−2.68 – 1.64)
Guinea – Bissau	2,772 (1,951 – 3,873)	396.03 (290.77 – 545.18)	1,920 (1,302 – 2,691)	141.06 (98.92 – 193.18)	−2.77 (−3.12 – 2.41)
Indonesia	572 (348 – 870)	0.37 (0.22 – 0.56)	517 (316 – 825)	0.18 (0.11 – 0.28)	−2.13 (−2.58 – 1.68)
Iran (Islamic Republic of)	404 (233 – 713)	0.9 (0.54 – 1.48)	128 (81 – 186)	0.15 (0.09 – 0.21)	−5.84 (−7.09 – 4.56)
Iraq	10,354 (5,676 – 18,877)	69.58 (41.43 – 119.79)	20,628 (8,958 – 43,218)	50.01 (23.25 – 104.51)	−1.28 (−1.36 – 1.2)
Jordan	187 (99 – 314)	7.42 (4.17 – 11.5)	429 (192 – 899)	3.63 (1.77 – 7.3)	−2.74 (−2.93 – 2.55)
Kenya	46,252 (25,510 – 85,602)	252.46 (155.49 – 451.91)	92,852 (46,821 – 177,918)	198.21 (108.02 – 371.91)	−0.94 (−1.06 – 0.81)
Lao People’s Democratic Republic	767 (508 – 1,104)	26.8 (17.68 – 38.71)	443 (255 – 709)	7.45 (4.26 – 11.56)	−4.32 (−4.59 – 4.04)
Lebanon	29 (15 – 50)	1.18 (0.61 – 1.92)	42 (20 – 75)	0.7 (0.35 – 1.26)	−1.46 (−1.59 – 1.34)
Liberia	9,934 (6,696 – 15,313)	492.71 (342.32 – 735.79)	6,238 (3,828 – 10,642)	136.4 (89.73 – 220.42)	−2.48 (−3.05 – 1.91)
Libya	2,936 (1,488 – 5,731)	76.52 (42 – 143.11)	4,228 (1,912 – 8,953)	55.86 (26.04 – 116.85)	−1.05 (−1.18 – 0.92)
Madagascar	34,998 (25,278 – 52,429)	351.96 (265.02 – 500.32)	35,772 (22,263 – 60,903)	147.43 (99.63 – 240.35)	−2.54 (−2.71 – 2.38)
Malawi	23,068 (16,573 – 34,795)	296.93 (220.95 – 426.83)	10,670 (8,070 – 14,618)	78.48 (60.02 – 102.3)	−4.26 (−4.93 – 3.59)
Mali	35,420 (26,177 – 52,183)	468.4 (361.42 – 654.92)	23,258 (16,456 – 33,253)	132.81 (101.15 – 178.35)	−3.91 (−4.38 – 3.43)
Mauritania	2,348 (1,438 – 3,789)	145.37 (92.81 – 220.8)	1,510 (800 – 2,677)	41.51 (23.84 – 68.07)	−3.13 (−3.51 – 2.74)
Mauritius	3,751 (1,814 – 7,675)	330.33 (163.3 – 663.95)	3,944 (1,826 – 8,125)	288.79 (132.34 – 602.02)	−0.37 (−0.4 – 0.33)
Morocco	4,317 (3,007 – 6,612)	21.01 (15.41 – 30.56)	4,531 (2,859 – 8,073)	12.03 (7.66 – 21.26)	−1.76 (−1.8 – 1.73)
Mozambique	22,788 (16,490 – 31,366)	213.83 (162.74 – 285.81)	25,232 (17,878 – 35,831)	116.98 (85.99 – 158.76)	−0.92 (−1.32 – 0.51)
Namibia	3,336 (2,059 – 5,502)	287.82 (186.07 – 451.18)	3,897 (2,051 – 7,378)	164.8 (92.27 – 302.33)	−2.01 (−2.3 – 1.72)
Niger	17,412 (12,937 – 24,072)	271.26 (205.24 – 362.97)	11,092 (8,323 – 14,497)	70.28 (52.88 – 90.89)	−4.22 (−4.64 – 3.8)
Nigeria	227,698 (146,216 – 363,626)	312.06 (210.35 – 484.38)	436,961 (231,147 – 795,508)	212.34 (118.63 – 384.47)	−1.52 (−1.66 – 1.37)
Oman	355 (185 – 706)	23.62 (13.01 – 42.14)	684 (272 – 1,539)	13.83 (6.05 – 31)	−1.52 (−1.65 – 1.39)
Philippines	14,510 (9,322 – 27,101)	27.35 (18.59 – 48.39)	12,200 (9,109 – 18,992)	11.73 (9.02 – 17.68)	−2.08 (−2.5 – 1.66)
Rwanda	23,627 (17,850 – 31,991)	464.76 (354.06 – 604.28)	7,610 (5,208 – 11,084)	73.31 (53.3 – 102.94)	−6.26 (−6.68 – 5.84)
Saint Lucia	0 (0 – 1)	0.33 (0.17 – 0.52)	1 (0 – 1)	0.38 (0.2 – 0.61)	0.16 (0.07 – 0.26)
Sao Tome and Principe	2 (1 – 3)	1.71 (0.86 – 2.61)	2 (1 – 4)	1.51 (0.71 – 2.62)	−0.51 (−0.67 – 0.35)
Saudi Arabia	3,061 (1,790 – 5,088)	24.92 (15.93 – 37.11)	4,953 (2,318 – 10,179)	12 (5.93 – 23.56)	−2.47 (−2.57 – 2.37)
Senegal	42,364 (28,800 – 64,905)	638.34 (440.94 – 961.77)	23,879 (14,503 – 40,855)	173.48 (111.82 – 280.96)	−3.73 (−4.18 – 3.29)
Sierra Leone	10,084 (7,086 – 14,771)	273.37 (198.77 – 392.86)	5,399 (3,801 – 7,460)	80.17 (57.86 – 109.45)	−3.8 (−4.64 – 2.96)
Somalia	17,570 (12,826 – 24,014)	330.63 (242.99 – 438.66)	23,358 (16,984 – 31,868)	187.03 (136.8 – 250.4)	−1.51 (−1.79 – 1.24)
South Africa	25,793 (14,159 – 46,068)	72.51 (41.15 – 129.38)	35,045 (17,271 – 67,750)	58.62 (29.42 – 112.6)	−0.63 (−0.99 – 0.26)
South Sudan	6,885 (4,943 – 9,767)	145.73 (106.33 – 200.18)	7,948 (5,145 – 12,785)	97.83 (67.72 – 144.35)	−1.4 (−1.54 – 1.25)
Sudan	12,604 (8,904 – 18,145)	76.23 (56.12 – 107.14)	5,746 (3,780 – 8,474)	16.67 (11.65 – 23.4)	−3.79 (−4.29 – 3.29)
Suriname	398 (234 – 733)	107.03 (64.54 – 190.6)	464 (222 – 938)	77.34 (36.85 – 156.48)	−1.07 (−1.19 – 0.96)
Syrian Arab Republic	2,060 (1,226 – 3,620)	21.65 (13.72 – 35.64)	1,907 (950 – 3,946)	13.37 (6.72 – 27.84)	−1.63 (−1.85 – 1.41)
Togo	10,878 (6,735 – 18,377)	376.26 (247.88 – 619.06)	5,940 (4,303 – 7,917)	94.19 (69 – 122.87)	−3.72 (−4.8 – 2.63)
Tunisia	1,041 (584 – 1,898)	14.13 (8.3 – 24.82)	1,253 (630 – 2,508)	10.11 (5.01 – 20.2)	−1.09 (−1.14 – 1.04)
Turkey	6,042 (4,342 – 8,744)	12.62 (9.34 – 17.32)	516 (350 – 751)	0.57 (0.39 – 0.82)	−9.03 (−9.83 – 8.22)
Uganda	67,186 (40,391 – 114,615)	508.27 (330.64 – 822.67)	54,091 (34,412 – 87,620)	159.98 (109.49 – 247.67)	−3.27 (−3.91 – 2.62)
United Republic of Tanzania	99,506 (67,076 – 156,354)	434.36 (305.18 – 661.3)	59,599 (37,695 – 99,816)	121.99 (82.74 – 190.56)	−3.68 (−4.25 – 3.11)
Venezuela (Bolivarian Republic of)	5,313 (3,165 – 9,606)	31.31 (20.05 – 53.68)	6,405 (3,429 – 12,421)	23.4 (12.17 – 45.41)	−0.88 (−1.01 – 0.74)
Yemen	15,716 (10,015 – 26,421)	168.32 (113.26 – 267.15)	8,250 (5,677 – 11,973)	35.16 (25.33 – 48.72)	−5.77 (−6.46 – 5.07)
Zambia	22,304 (15,889 – 33,090)	347.85 (260.5 – 488.52)	24,483 (15,190 – 41,392)	148.32 (98.95 – 234.21)	−2.56 (−3.02 – 2.1)
Zimbabwe	11,153 (6,767 – 19,919)	124.83 (82.51 – 207.64)	12,696 (9,113 – 17,762)	105.81 (78.11 – 141.44)	0.33 (−0.41 – 1.08)

UI: uncertainty intervals; CI: confidence interval; SDI: socio-demographic index; EAPC: estimated annual percent change.

**Table 3:** Number of prevalence cases and the age-standardized prevalence rate (ASPR) attributable to schistosomiasis in 1990 and 2021, and trends from 1990 to 2021 globally.

Characteristics	Number of prevalence cases (95% UI) in 1990	The age – standardized prevalence rate/100,000 (95% UI) in 1990	Number of prevalence cases (95% UI) in 2021	The age – standardized prevalence rate/100,000 (95% UI) in 2021	EAPC (95% CI)
Global	136,534,484 (111,224,402 – 162,733,457)	2,473.94 (2,012.9 – 2,957.52)	151,376,744 (109,062,891 – 198,666,396)	1,914.3 (1,378.92 – 2,510.85)	−0.63 (−0.95 – 0.31)
**Age**					
<5 years	339,677 (109,415 – 796,821)	54.79 (17.65 – 128.53)	110,109 (24,536 – 328,846)	16.73 (3.73 – 49.96)	−0.56 (−2.64 – 1.57)
5–9 years	11,683,999 (8,342,752 – 15,306,359)	2,002.29 (1,429.7 – 2,623.06)	4,434,015 (1,837,686 – 7,971,911)	645.37 (267.47 – 1,160.3)	−1.82 (−2.96 – 0.68)
10–14 years	20,794,356 (14,984,302 – 27,485,736)	3,881.84 (2,797.23 – 5,130.97)	16,183,562 (8,525,195 – 27,442,108)	2,427.65 (1,278.84 – 4,116.51)	−0.52 (−1.14 – 0.1)
15–19 years	20,517,353 (13,677,150 – 27,968,793)	3,950.02 (2,633.14 – 5,384.58)	24,230,157 (13,249,191 – 37,305,907)	3,883.16 (2,123.34 – 5,978.7)	0.15 (−0.1 – 0.4)
20–24 years	18,920,436 (12,306,647 – 25,711,086)	3,844.93 (2,500.9 – 5,224.9)	22,834,057 (12,491,952 – 33,915,497)	3,823.79 (2,091.9 – 5,679.48)	−0.16 (−0.42 – 0.1)
25–29 years	16,060,789 (10,763,812 – 21,934,747)	3,628.58 (2,431.84 – 4,955.66)	19,727,024 (11,307,399 – 29,708,296)	3,352.98 (1,921.91 – 5,049.48)	−0.39 (−0.69 – 0.08)
30–34 years	12,831,947 (8,394,478 – 17,302,798)	3,329.32 (2,177.99 – 4,489.31)	16,776,746 (9,535,090 – 25,310,938)	2,775.4 (1,577.4 – 4,187.22)	−0.58 (−0.85 – 0.31)
35–39 years	10,506,291 (7,542,309 – 14,213,943)	2,982.67 (2,141.21 – 4,035.25)	13,273,720 (8,128,458 – 20,226,144)	2,366.65 (1,449.27 – 3,606.23)	−0.73 (−0.94 – 0.53)
40–44 years	7,538,311 (5,100,595 – 10,588,759)	2,631.34 (1,780.43 – 3,696.14)	10,115,186 (5,719,744 – 16,106,584)	2,022.02 (1,143.38 – 3,219.7)	−0.97 (−1.14 – 0.79)
45–49 years	5,541,172 (4,098,024 – 7,426,534)	2,386.43 (1,764.9 – 3,198.4)	7,688,649 (4,982,283 – 11,275,312)	1,623.77 (1,052.21 – 2,381.25)	−1.36 (−1.49 – 1.24)
50–54 years	4,025,375 (3,113,357 – 5,166,745)	1,893.66 (1,464.62 – 2,430.59)	5,679,462 (3,976,540 – 7,969,169)	1,276.5 (893.76 – 1,791.13)	−1.6 (−1.79 – 1.42)
55–59 years	2,837,103 (1,984,506 – 3,989,501)	1,531.91 (1,071.55 – 2,154.16)	3,929,407 (2,460,643 – 6,022,878)	992.96 (621.8 – 1,521.97)	−1.72 (−1.95 – 1.49)
60–64 years	2,010,329 (1,269,202 – 3,062,589)	1,251.69 (790.24 – 1,906.86)	2,484,457 (1,338,765 – 4,267,598)	776.28 (418.3 – 1,333.42)	−1.79 (−2.02 – 1.55)
65–69 years	1,380,385 (956,514 – 1,971,191)	1,116.73 (773.82 – 1,594.69)	1,795,010 (1,110,187 – 2,795,388)	650.74 (402.47 – 1,013.4)	−1.84 (−2.06 – 1.62)
70–74 years	844,755 (610,647 – 1,232,446)	997.81 (721.28 – 1,455.74)	1,066,643 (711,268 – 1,660,829)	518.19 (345.55 – 806.86)	−1.94 (−2.05 – 1.83)
75–79 years	432,939 (277,644 – 670,836)	703.33 (451.05 – 1,089.81)	575,544 (323,801 – 953,444)	436.4 (245.52 – 722.94)	−1.95 (−2.1 – 1.81)
80–84 years	185,300 (109,034 – 297,524)	523.8 (308.21 – 841.04)	294,023 (152,904 – 515,104)	335.71 (174.58 – 588.13)	−1.75 (−1.99 – 1.52)
85–89 years	65,636 (41,388 – 103,069)	434.35 (273.89 – 682.08)	127,748 (76,439 – 208,725)	279.4 (167.18 – 456.51)	−1.47 (−1.71 – 1.24)
90–94 years	15,387 (9,091 – 27,655)	359.07 (212.15 – 645.37)	40,547 (22,713 – 73,580)	226.65 (126.96 – 411.31)	−1.32 (−1.5 – 1.15)
95+ years	2,945 (1,262 – 6,388)	289.24 (123.95 – 627.48)	10,679 (4,549 – 23,026)	195.93 (83.47 – 422.48)	−1.06 (−1.13 – 1)
**Sex**					
Female	62,486,337 (50,400,556 – 75,171,103)	2,284.05 (1,841.1 – 2,755)	70,079,922 (496,897,38 – 930,227,15)	1,795.23 (1,269.64 – 2,372.76)	−0.58 (−0.9 – 0.27)
Male	74,048,147 (60,698,477 – 87,372,607)	2,662.98 (2,186.16 – 3,142.23)	81,296,823 (592,935,51 – 1,055,654,20)	2,030.97 (1,485.87 – 2,643.59)	−0.67 (−1.01 – 0.34)
**SDI region**					
High-middle SDI	2,401,297 (1,706,082 – 3,166,603)	214.73 (152.87 – 285.23)	1,574,082 (1,094,337 – 2,176,784)	123.11 (84.59 – 172.95)	−1.92 (−2.23 – 1.61)
High SDI	255,625 (134,036 – 352,520)	30.3 (15.92 – 41.7)	603,224 (298,639 – 953,277)	59.47 (28.78 – 94.51)	2.21 (2.01 – 2.4)
Low-middle SDI	43,938,906 (37,643,175 – 50,415,633)	3,825.31 (3,295.92 – 4,386.77)	49,888,984 (362,577,62 – 652,392,16)	2,461.03 (1,805.47 – 3,212.18)	−1.73 (−2 – 1.46)
Low SDI	60,247,237 (48,744,464 – 72,460,170)	12,588.24 (10,118.8 – 15,263.09)	63,427,146 (450,572,51 – 841,449,40)	5,832.77 (4,227.86 – 7,675.46)	−2.05 (−2.56 – 1.53)
Middle SDI	29,622,291 (22,672,657 – 38,350,644)	1,701.03 (1,310.39 – 2,185.2)	35,791,267 (261,608,93 – 465,838,41)	1,413.61 (1,029.41 – 1,822.78)	−0.62 (−0.65 – 0.59)
**GBD region**					
*Advanced health system*	614,585 (402,311 – 876,291)	47.18 (30.56 – 67.14)	698,802 (360,261 – 1,098,092)	49.36 (24.6 – 78.47)	−0.74 (−1.48 – 0.01)
Africa	110,732,176 (91,103,993 – 131,173,338)	18,495.51 (15,166.27 – 22,183.82)	127,535,372 (913,563,30 – 167,805,780)	9,461.76 (6,900.58 – 12,501.22)	−2.03 (−2.41 – 1.66)
African Region	90,895,195 (71,791,673 – 110,370,208)	19,008.75 (14,997.01 – 23,545.76)	123,854,132 (887,595,38 – 162,827,981)	11,152.81 (8,131.01 – 14,756.35)	−1.52 (−1.86 – 1.17)
America	5,625,944 (4,136,699 – 7,273,928)	766.19 (567.18 – 990.27)	7,790,481 (5,690,289 – 10,423,476)	729.36 (526.74 – 971.36)	−0.04 (−0.2 – 0.11)
Asia	19,781,686 (15,357,021 – 27,602,917)	620.11 (487.32 – 860.22)	15,958,850 (12,217,390 – 21,088,536)	325.39 (247.69 – 430.51)	−2.12 (−2.18 – 2.06)
*Basic health system*	45,854,262 (38,992,575 – 54,331,966)	1,923.71 (1,649.94 – 2,291.96)	30,833,383 (23,004,041 – 40,257,016)	946.73 (698.59 – 1,220.16)	−2.55 (−2.82 – 2.29)
Caribbean	810,125 (534,886 – 1,156,634)	2,207.91 (1,491.08 – 3,140.13)	1,246,510 (829,969 – 1,802,980)	2,567.38 (1,709.77 – 3,683.91)	0.48 (0.45 – 0.51)
Central Africa	12,164,438 (9,574,316 – 14,776,444)	19,626.48 (15,444.41 – 23,812.17)	11,631,815 (8,035,099 – 15,843,336)	7,379.21 (5,189.5 – 10,289.32)	−1.87 (−2.63 – 1.1)
Central Latin America	565,071 (443,618 – 701,457)	343.56 (273.82 – 431.27)	744,419 (509,565 – 1,057,607)	280.79 (192.05 – 399.07)	−0.1 (−0.3 – 0.11)
Central Sub-Saharan Africa	9,687,350 (7,446,362 – 11,891,045)	19,116.62 (14,747.57 – 23,524.35)	10,268,753 (7,147,341 – 13,878,619)	7,947.47 (5,702.6 – 11,151.88)	−1.43 (−2.07 – 0.78)
Commonwealth High Income	4,253 (1,189 – 8,135)	3.78 (1.07 – 7.44)	6,354 (1,868 – 11,850)	4.57 (1.35 – 8.65)	0.64 (0.43 – 0.85)
Commonwealth Low Income	21,159,218 (16,606,142 – 26,360,319)	10,622.4 (8,305.55 – 13,177.53)	19,958,412 (13,990,359 – 27,182,551)	4,948.86 (3,510.13 – 6,703.06)	−2 (−2.49 – 1.52)
Commonwealth Middle Income	29,286,222 (21,798,246 – 37,396,618)	2,541.37 (1,907.45 – 3,274.3)	60,151,949 (433,506,26 – 789,617,66)	2,672.25 (1,945.87 – 3,489.3)	0.02 (−0.14 – 0.18)
East Asia	15,713,519 (12,373,489 – 21,733,893)	1,223.58 (973.26 – 1,695.55)	11,459,581 (8,953,887 – 15,054,895)	733.95 (559.29 – 955.91)	−1.62 (−1.77 – 1.48)
East Asia & Pacific – WB	16,986,751 (13,229,342 – 24,685,143)	870.66 (686.16 – 1,254.04)	12,097,001 (9,418,777 – 16,452,634)	473.81 (362.84 – 641.24)	−1.81 (−1.88 – 1.75)
Eastern Africa	32,750,686 (26,701,153 – 39,339,600)	19,908.61 (16,229.06 – 23,797.34)	44,203,214 (321,462,26 – 571,482,01)	11,923.37 (8,834.03 – 15,419.27)	−1.55 (−1.83 – 1.28)
Eastern Mediterranean Region	22,631,916 (21,039,510 – 24,472,494)	6,281.24 (5,840.52 – 6,774.15)	7,543,089 (5,303,981 – 10,745,853)	985.82 (710.49 – 1,392.89)	−6.41 (−7.3 – 5.51)
Eastern Sub-Saharan Africa	35,955,363 (28,983,377 – 43,615,294)	20,155.1 (16,221.07 – 24,513.01)	46,949,044 (33,903,446 – 61,048,520)	11,617.62 (8,557.99 – 15,093.06)	−1.64 (−1.95 – 1.33)
Latin America & Caribbean – WB	5,625,944 (4,136,699 – 7,273,928)	1,289.71 (965.11 – 1,671.56)	7,790,481 (5,690,289 – 10,423,476)	1,116.27 (816.28 – 1,485.49)	−0.35 (−0.53 – 0.17)
*Limited health system*	70,395,906 (55,697,691 – 85,625,326)	4,515.26 (3,556.93 – 5,561.58)	105,609,322 (76,017,154 – 138,783,527)	3,465.04 (2,526.64 – 4,499.73)	−0.86 (−1.12 – 0.6)
Middle East & North Africa – WB	21,276,166 (19,858,078 – 22,873,959)	8,519.42 (7,966.98 – 9,134.69)	6,935,877 (4,909,950 – 9,913,486)	1,402.61 (1,000.13 – 1,982.74)	−6.62 (−7.54 – 5.69)
*Minimal health system*	19,600,603 (15,611,472 – 23,541,714)	15,489.78 (12,333.04 – 18,731.15)	14,143,196 (9,706,633 – 19,388,077)	4,562.09 (3,192.75 – 6,273.6)	−2.66 (−3.51 – 1.8)
North Africa and Middle East	22,391,513 (20,849,802 – 24,172,229)	6,744.85 (6,289.09 – 7,239.6)	7,289,692 (5,158,092 – 10,390,474)	1,127.85 (805.09 – 1,597.26)	−6.4 (−7.3 – 5.5)
Northern Africa	18,620,783 (17,783,564 – 19,503,508)	15,572.7 (14,885.84 – 16,317.27)	3,194,927 (2,297,969 – 4,539,483)	1,509.2 (1,096.12 – 2,119.55)	−8.16 (−9.45 – 6.86)
Region of the Americas	5,625,944 (4,136,699 – 7,273,928)	766.19 (567.18 – 990.27)	7,790,481 (5,690,289 – 10,423,476)	729.36 (526.74 – 971.36)	−0.04 (−0.2 – 0.11)
South-East Asia Region	20,798 (13,138 – 58,389)	1.65 (1.04 – 4.59)	8,805 (5,068 – 24,869)	0.4 (0.23 – 1.11)	−2.95 (−3.99 – 1.9)
Southeast Asia	1,647,269 (1,137,708 – 3,155,330)	356.12 (252.37 – 683.2)	1,052,615 (717,566 – 1,797,568)	142.81 (97.28 – 244.13)	−2.1 (−2.7 – 1.5)
Southern Africa	8,547,928 (6,391,607 – 10,953,659)	9,420.42 (7,126.23 – 12,225.52)	10,095,872 (7,198,300 – 13,817,354)	5,603.26 (4,057.96 – 7,690.46)	−1.06 (−1.43 – 0.69)
Southern Sub-Saharan Africa	4,030,543 (3,003,495 – 5,118,048)	7,718.27 (5,839.42 – 10,024.38)	4,989,541 (3,609,448 – 6,699,970)	5,950.95 (4,379.21 – 7,964.84)	−0.62 (−0.87 – 0.37)
Sub-Saharan Africa – WB	92,250,946 (72,948,645 – 111,946,003)	19,221.42 (15,181.77 – 23,783.58)	124,461,344 (891,335,33 – 1,636,585,43)	11,082.39 (8,071.8 – 14,688.09)	−1.55 (−1.91 – 1.2)
Tropical Latin America	4,278,179 (3,113,210 – 5,540,455)	2,785.6 (2,076.29 – 3,602.72)	5,841,735 (4,317,716 – 7,647,499)	2,395.45 (1,751.94 – 3,105.58)	−0.41 (−0.68 – 0.13)
Western Africa	38,648,341 (30,097,446 – 47,829,871)	23,934.72 (18,609.52 – 30,047.45)	58,409,543 (419,405,25 – 769,278,37)	14,308.56 (10,299.83 – 18,892.56)	−1.72 (−2.04 – 1.4)
Western Pacific Region	16,965,953 (13,214,249 – 24,631,109)	1,044.5 (821.93 – 1,504.06)	12,088,196 (9,414,150 – 16,428,215)	592.86 (450.86 – 802.97)	−1.69 (−1.76 – 1.62)
Western Sub-Saharan Africa	41,455,550 (32,488,638 – 51,087,192)	23,185.69 (18,122.45 – 29,010.43)	61,534,854 (440,350,20 – 813,380,05)	13,433.55 (9,651.8 – 17,812.08)	−1.78 (−2.14 – 1.42)
World Bank High Income	293,288 (161,611 – 405,632)	29.54 (16.02 – 41.18)	705,155 (366,486 – 1,106,552)	62.3 (31.3 – 99.02)	2.46 (2.28 – 2.65)
World Bank Low Income	43,240,690 (34,953,558 – 52,297,136)	14,804.5 (11,871.73 – 17,846.38)	44,357,738 (318,106,93 – 579,479,87)	6,762.61 (4,946.01 – 8,805.95)	−1.96 (−2.47 – 1.44)
World Bank Lower Middle Income	66,334,532 (55,410,717 – 78,297,346)	3,315.8 (2,756.85 – 3,917.03)	78,762,595 (56,451,244 – 104,003,482)	2,170.98 (1,563.31 – 2,859.46)	−1.57 (−1.88 – 1.26)
World Bank Upper Middle Income	26,596,845 (20,583,159 – 33,893,558)	1,273.43 (999.25 – 1,620.4)	27,459,214 (20,686,533 – 35,388,387)	1,057.79 (777.5 – 1,355.31)	−0.5 (−0.63 – 0.37)
**Country**					
Algeria	79,041 (48,406 – 120,214)	331.85 (205.73 – 498.52)	145,707 (88,394 – 222,530)	327.23 (199.37 – 502.68)	−0.03 (−0.06 – 0)
Angola	1,102,833 (783,584 – 1,551,404)	12,030.57 (8,874.95 – 16,478.98)	1,890,750 (1,301,321 – 2,794,168)	6,431.53 (4,651.67 – 9,269.21)	−1.22 (−1.54 – 0.9)
Antigua and Barbuda	4,253 (1,189 – 8,135)	6,850.91 (1,935.35 – 12,851.79)	6,354 (1,868 – 11,850)	6,632.58 (1,949.93 – 12,500.73)	−0.07 (−0.09 – 0.05)
Benin	1,358,189 (1,114,987 – 1,584,561)	30,390.15 (25,258.17 – 35,175.58)	1,333,047 (896,247 – 1,897,618)	10,643.3 (7,435.21 – 14,765.79)	−1.73 (−2.88 – 0.58)
Botswana	253,878 (171,495 – 341,153)	19,238.57 (13,492.76 – 24,794.76)	370,185 (257,446 – 499,927)	14,369.98 (10,204.5 – 19,243.96)	−0.2 (−0.37 – 0.02)
Brazil	4,278,179 (3,113,210 – 5,540,455)	2,855.26 (2,128 – 3,692.78)	5,841,735 (4,317,716 – 7,647,499)	2,472.93 (1,809.89 – 3,205.39)	−0.38 (−0.66 – 0.11)
Burkina Faso	2,176,021 (1,594,657 – 2,726,965)	23,528.74 (18,027.35 – 28,429.99)	250,291 (185,989 – 323,773)	1,306.63 (972.71 – 1,675.83)	−8.74 (−10.73 – 6.72)
Burundi	914,142 (615,987 – 1,279,654)	18,126.1 (12,702.71 – 25,098.5)	251,568 (136,863 – 422,652)	2,149.42 (1,170.67 – 3,579.64)	−5 (−6.54 – 3.43)
Cambodia	9,648 (2,962 – 42,084)	104.03 (32.03 – 454.57)	278 (74 – 1,279)	1.58 (0.43 – 7.32)	−13.05 (−15.16 – 10.87)
Cameroon	2,294,299 (1,973,579 – 2,673,805)	22,783.66 (19,459.75 – 26,789.28)	2,505,766 (1,683,224 – 3,662,303)	8,184.22 (5,709.62 – 11,642.08)	−2.98 (−3.93 – 2.03)
Central African Republic	363,176 (279,931 – 437,876)	15,118.95 (11,850.07 – 18,113.79)	552,996 (391,671 – 723,023)	10,629.64 (7,563.91 – 13,711.6)	−0.51 (−0.83 – 0.2)
Chad	371,474 (258,837 – 515,642)	7,206.94 (5,057.6 – 9,968.3)	496,477 (331,619 – 728,509)	3,245.69 (2,258.36 – 4,647.5)	−0.59 (−1.32 – 0.15)
China	15,713,519 (12,373,489 – 21,733,893)	1,266.48 (1,007.53 – 1,754.97)	11,459,581 (8,953,887 – 15,054,895)	761.32 (579.99 – 991.06)	−1.62 (−1.76 – 1.47)
Congo	394,371 (320,605 – 482,534)	16,990.92 (13,807.91 – 20,661.51)	470,062 (325,868 – 688,354)	8,617.34 (6,103.38 – 12,352.24)	−0.21 (−1.06 – 0.65)
Saint Helena	3,489,926 (2,769,587 – 4,228,048)	28,361.48 (22,238.03 – 35,090.42)	1,641,642 (1,126,321 – 2,407,838)	6,130.95 (4,316.89 – 8,595.5)	−4 (−4.75 – 3.23)
Democratic Republic of the Congo	7,591,223 (5,768,881 – 9,348,161)	21,538.54 (16,660.25 – 26,596.43)	7,196,689 (5,019,931 – 9,767,646)	8,382.85 (5,966.47 – 11,608.92)	−1.51 (−2.23 – 0.78)
Djibouti	510 (5 – 3,674)	123.73 (1.18 – 854.8)	1,450 (14 – 10,321)	110.39 (1.06 – 775.22)	−0.25 (−0.33 – 0.18)
Dominican Republic	738,375 (485,189 – 1,067,144)	10,388.4 (7,006.13 – 14,786.47)	1,144,573 (760,506 – 1,655,316)	9,923.69 (6,620.53 – 14,219.8)	−0.17 (−0.19 – 0.15)
Egypt	17,700,500 (16,946,837 – 18,538,076)	32,188.27 (30,918.82 – 33,648.66)	1,823,345 (1,347,602 – 2,570,424)	1,759.27 (1,316.97 – 2,440.85)	−9.91 (−11.55 – 8.23)
Equatorial Guinea	11,979 (9,473 – 14,323)	2,961.3 (2,336.75 – 3,562.42)	45,553 (28,122 – 66,633)	2,924.48 (1,885.73 – 4,171.64)	−0.16 (−0.2 – 0.12)
Eritrea	296,075 (208,535 – 393,746)	9,258.46 (6,637.07 – 12,196.85)	148,932 (99,781 – 215,431)	2,242.21 (1,552.11 – 3,221.72)	−2.53 (−3.57 – 1.47)
Eswatini	25,919 (16,926 – 38,206)	3,474.71 (2,380.23 – 5,051.94)	29,416 (19,344 – 43,158)	2,398.86 (1,626.43 – 3,465.02)	−0.52 (−0.78 – 0.26)
Ethiopia	97,137,300 (80,649,050 – 1,128,980,100)	20,521.27 (17,153.86 – 23,799)	20,950,596 (15,643,895 – 26,487,899)	20,629.5 (15,623.73 – 25,837.5)	−0.29 (−0.41 – 0.16)
Gabon	223,767 (157,904 – 285,053)	23,394.56 (16,965.25 – 29,391.93)	112,703 (76,468 – 167,107)	6,168.94 (4,246.72 – 9,135.21)	−2.05 (−2.91 – 1.17)
Gambia	78,551 (53,266 – 113,641)	8,875.08 (6,204.42 – 12,469.1)	96,848 (64,126 – 143,258)	4,163.82 (2,883.56 – 5,903.51)	−0.78 (−1.41 – 0.15)
Ghana	56,077,900 (41,473,820 – 70,346,790)	38,421.82 (29,323.38 – 48,280.48)	6,626,574 (4,498,970 – 9,145,147)	19,321.32 (13,477.54 – 26,646.31)	−2.11 (−2.62 – 1.6)
Guinea	1,082,898 (831,246 – 1,333,122)	18,990.11 (14,527.44 – 24,221.14)	695,679 (473,100 – 991,015)	5,601.23 (3,859.55 – 7,860.35)	−2.16 (−3 – 1.3)
Guinea-Bissau	91,176 (62,760 – 123,654)	10,113.49 (7,276.1 – 13,609.45)	48,170 (31,919 – 71,279)	2,438.12 (1,675.71 – 3,427.46)	−2.33 (−3.3 – 1.35)
Indonesia	20,798 (13,138 – 58,389)	11.34 (7.17 – 31.58)	8,805 (5,068 – 24,869)	2.9 (1.68 – 8.19)	−2.68 (−3.74 – 1.61)
Iran (Islamic Republic of)	35,240 (21,826 – 53,634)	66.27 (42.43 – 97.57)	1,436 (714 – 2,582)	1.52 (0.75 – 2.73)	−12.31 (−15.75 – 8.73)
Iraq	1,001,594 (667,161 – 1,434,979)	6,001.1 (4,144.44 – 8,479.25)	2,578,519 (1,760,216 – 3,737,283)	6,070.65 (4,215.96 – 8,609.98)	0.07 (0.04 – 0.1)
Jordan	12,169 (5,402 – 23,316)	348.37 (157.4 – 654.03)	47,294 (21,376 – 88,569)	361.11 (163.93 – 674.95)	0.08 (0.07 – 0.1)
Kenya	47,528,730 (34,704,020 – 62,147,680)	22,714.64 (16,821.48 – 29,647.42)	10,514,322 (7,592,860 – 13,770,716)	20,536.94 (15,062.14 – 26,748.39)	−0.94 (−1.29 – 0.58)
Lao People’s Democratic Republic	10,931 (6,586 – 26,503)	295.66 (179.31 – 724.82)	1,038 (558 – 2,637)	13.43 (7.29 – 33.64)	−8.49 (−10.41 – 6.52)
Lebanon	111 (0 – 2,617)	3.83 (0 – 91.65)	227 (0 – 5,230)	3.85 (0 – 88.24)	−0.01 (−0.06 – 0.03)
Liberia	655,981 (476,602 – 850,035)	29,922.38 (22,217.37 – 39,625.69)	430,345 (294,045 – 616,363)	7,971.37 (5,587.38 – 11,221.56)	−1.54 (−2.46 – 0.61)
Libya	353,939 (264,192 – 453,344)	8,602.63 (6,562.38 – 10,730.56)	557,731 (384,483 – 802,219)	7,250.48 (5,041.99 – 10,321.29)	−0.6 (−0.68 – 0.53)
Madagascar	22,224,030 (19,880,260 – 26,316,080)	19,568.27 (17,542.55 – 22,530.5)	2,896,859 (1,994,438 – 3,930,358)	10,153.64 (7,095.42 – 13,765.96)	−1.93 (−2.16 – 1.71)
Malawi	12,201,200 (8,482,850 – 16,726,870)	13,479.82 (9,628.01 – 18,527.59)	300,432 (197,643 – 446,707)	1,571.4 (1,079.55 – 2,245.08)	−5.7 (−6.95 – 4.43)
Mali	19,578,760 (17,682,340 – 21,563,280)	23,270.59 (21,057.09 – 25,666.67)	947,482 (559,134 – 1,467,911)	3,994.83 (2,474.54 – 5,877.98)	−5.22 (−6.38 – 4.05)
Mauritania	140,063 (77,315 – 204,938)	7,241.23 (4,099.96 – 10,724.61)	122,349 (65,011 – 191,139)	2,930.81 (1,649.22 – 4,533.1)	−1.71 (−2.4 – 1.02)
Mauritius	371,655 (257,872 – 496,837)	32,067.77 (22,529.77 – 42,577.78)	413,727 (292,610 – 548,453)	30,746.27 (21,636.25 – 41,213.11)	−0.11 (−0.12 – 0.09)
Morocco	249,512 (160,011 – 365,599)	1,020.32 (674.82 – 1,473.19)	394,929 (255,574 – 576,297)	1,024.07 (662.91 – 1,494.41)	−0.04 (−0.06 – 0.02)
Mozambique	945,345 (677,432 – 1,229,821)	7,731.35 (5,619.37 – 9,973.23)	983,784 (661,386 – 1,437,565)	3,337.88 (2,298.48 – 4,621.71)	−1.36 (−2.04 – 0.67)
Namibia	265,484 (197,010 – 329,695)	20,094.47 (15,104.08 – 25,061.46)	417,826 (296,731 – 545,369)	16,440.77 (11,858.73 – 21,480.78)	−0.67 (−0.82 – 0.52)
Niger	783,003 (576,715 – 996,932)	10,981.35 (8,145.83 – 14,182.67)	143,871 (93,237 – 217,283)	679.39 (471.6 – 980.31)	−6.63 (−8.28 – 4.94)
Nigeria	167,764,900 (119,671,740 – 2,224,236,300)	20,901.76 (15,164.07 – 27,970.69)	44,185,123 (31,953,461 – 57,552,999)	20,432.61 (14,906.45 – 26,836.63)	−0.37 (−0.57 – 0.18)
Oman	33,409 (17,791 – 58,383)	1,776.86 (1,005.01 – 3,078.77)	95,577 (51,121 – 164,669)	1,810.38 (1,015.82 – 3,070.71)	0.13 (0.09 – 0.17)
Philippines	12,318,540 (8,249,680 – 26,719,170)	2,043.21 (1,393.44 – 4,408.04)	627,299 (389,644 – 1,384,935)	535.21 (336.5 – 1,178.35)	−3.12 (−3.92 – 2.32)
Rwanda	888,354 (585,770 – 1,279,694)	13,493.38 (9,256.13 – 19,115.94)	314,740 (207,591 – 456,654)	2,369.55 (1,601.76 – 3,347.58)	−3.23 (−4.41 – 2.03)
Sao Tome and Principe	5 (0 – 0)	5.09 (0 – 0)	2 (0 – 0)	0.78 (0 – 0)	−3.79 (−5.03 – 2.54)
Saudi Arabia	255,625 (134,036 – 352,520)	1,602.85 (846.55 – 2,182.71)	603,224 (298,639 – 953,277)	1,364.04 (687.19 – 2,130.5)	−0.58 (−0.69 – 0.46)
Senegal	30,854,280 (27,866,910 – 33,293,910)	43,044.76 (39,175.02 – 46,587.95)	1,712,953 (1,149,415 – 2,464,902)	11,062.95 (7,568.96 – 15,692.71)	−3.98 (−4.64 – 3.31)
Sierra Leone	647,751 (584,433 – 717,805)	16,238.99 (14,559.87 – 18,017.31)	162,611 (104,628 – 234,861)	1,891.69 (1,271.27 – 2,643.4)	−7.01 (−8.69 – 5.29)
Somalia	656,733 (435,997 – 927,751)	9,001.49 (6,238.91 – 12,180.52)	404,453 (261,786 – 602,728)	2,041.75 (1,366.66 – 2,874.32)	−2.34 (−3.37 – 1.31)
South Africa	24,416,200 (17,686,690 – 32,444,790)	6,628.37 (4,832.34 – 8,844.13)	36,719,67 (26,760,25 – 49,081,58)	6,069.07 (4,476.19 – 8,047.24)	−0.02 (−0.27 – 0.22)
South Sudan	365,519 (295,580 – 442,710)	6,299.66 (5,036.98 – 7,669)	527,146 (338,513 – 768,747)	5,352.92 (3,578.16 – 7,379.4)	−0.54 (−0.61 – 0.46)
Sudan	778,058 (649,558 – 932,668)	3,968.85 (3,331.61 – 4,731.89)	348,466 (224,244 – 520,670)	812.12 (538.4 – 1,180.09)	−2.76 (−3.79 – 1.72)
Suriname	40,065 (26,326 – 52,542)	10,070.41 (6,646.52 – 13,233.6)	53,401 (31,954 – 77,140)	9,002.65 (5,388.28 – 13,071.63)	−0.34 (−0.41 – 0.27)
Syrian Arab Republic	163,929 (97,982 – 266,606)	1,442.66 (878.26 – 2,329.46)	215,201 (130,363 – 341,965)	1,480.61 (905.43 – 2,374.33)	0.09 (0.07 – 0.1)
Togo	8,572,600 (5,989,330 – 11,403,900)	25,943.4 (18,476.94 – 35,396.36)	134,907 (88,147 – 199,901)	1,713.23 (1,124.42 – 2,518.3)	−6.44 (−8.38 – 4.45)
Tunisia	97,728 (58,041 – 152,538)	1,197.12 (716.33 – 1,840.78)	150,867 (89,574 – 230,072)	1,232.77 (729.9 – 1,876.6)	0.15 (0.12 – 0.17)
Uganda	58,779,450 (43,693,990 – 74,659,320)	39,228.04 (29,906.96 – 49,191.44)	3,607,459 (2,428,766 – 5,183,661)	8,818 (6,290.03 – 12,423.79)	−3.13 (−4.01 – 2.25)
United Republic of Tanzania	68,268,300 (55,958,080 – 80,555,830)	26,761.76 (21,884.54 – 32,125.55)	4,075,065 (2,735,277 – 5,843,824)	7,188.2 (4,938.64 – 10,178.78)	−3.65 (−4.49 – 2.81)
Venezuela (Bolivarian Republic of)	565,071 (443,618 – 701,457)	2,946.66 (2,351.14 – 3,687.33)	744,419 (509,565 – 1,057,607)	2,776.32 (1,889.77 – 3,938.28)	−0.06 (−0.09 – 0.03)
Yemen	12,928,570 (9,331,800 – 17,080,530)	11,467.1 (8,277.31 – 15,288.73)	320,370 (210,007 – 464,344)	999.21 (682.08 – 1,392.74)	−7.82 (−9.2 – 6.41)
Zambia	12,490,870 (9,995,020 – 15,112,050)	16,169.15 (13,134.04 – 19,792.58)	1,931,366 (1,317,712 – 2,696,688)	9,966.6 (7,069.46 – 13,629.08)	−0.54 (−1.01 – 0.06)
Zimbabwe	10,436,430 (8,187,200 – 12,645,010)	9,804.7 (7,748.71 – 12,238.39)	500,147 (338,419 – 751,352)	3,170.34 (2,169.77 – 4,593.29)	−3.36 (−4.19 – 2.52)

UI: uncertainty intervals; CI: confidence interval; SDI: socio-demographic index; EAPC: estimated annual percent change.

**Figure 1:**
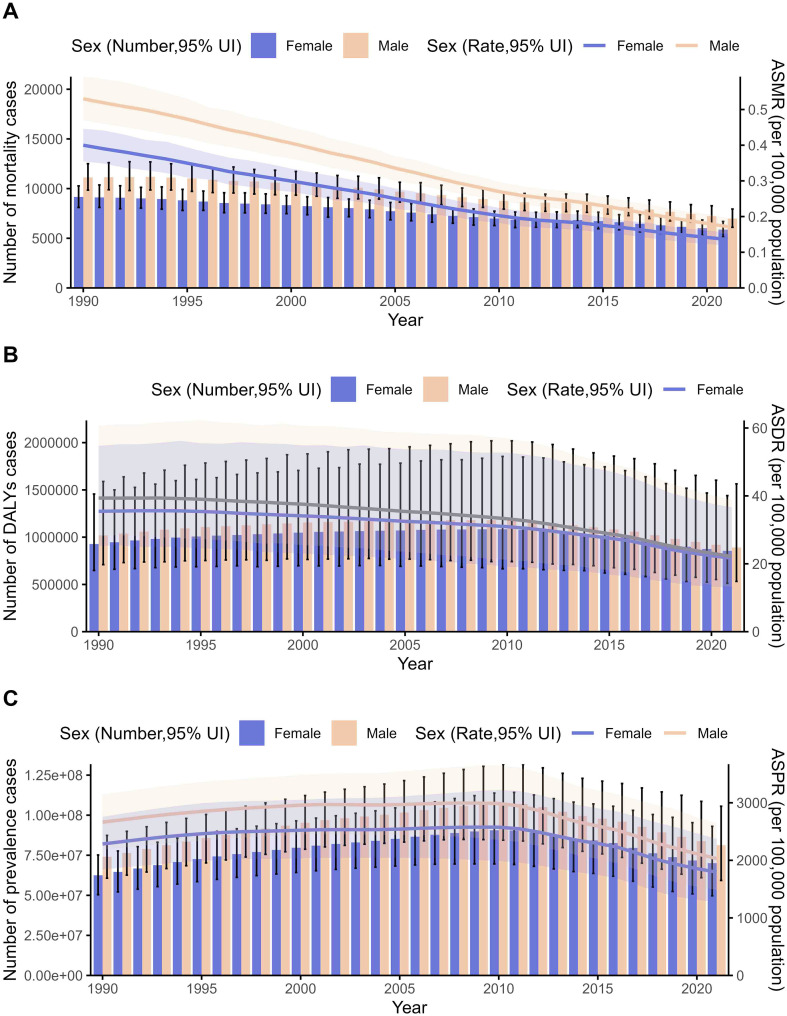
Global trends in the disease burden of schistosomiasis from 1990 to 2021. (A) Trends in number of deaths and ASMR. (B) Trends in DALYs and ASDR. (C) Trends in prevalence numbers and ASPR. Abbreviations: ASMR, Age-standardized Mortality Rate; ASDR, Age-Standardized DALY Rate; ASPR, Age-Standardized Prevalence Rate. DALYs, disability-adjusted life years.

Between 1990 and 2021, trends in the number of deaths, ASMR, DALYs, ASDR, prevalence number, and ASPR were also reported by the GBD 2021. Specifically, the number of deaths and ASMR showed a downward trend year by year, while the DALYs, ASDR, prevalence number (despite an overall increase), and ASPR exhibited an upward trend, followed by a decline (Fig. 1). Detailed annual changes and EAPCs with 95% confidence intervals (CIs) for these indicators are provided in Tables 1–3, respectively.

### Subgroup analysis in 2021 (based on GBD 2021 data)

**Figure 2:**
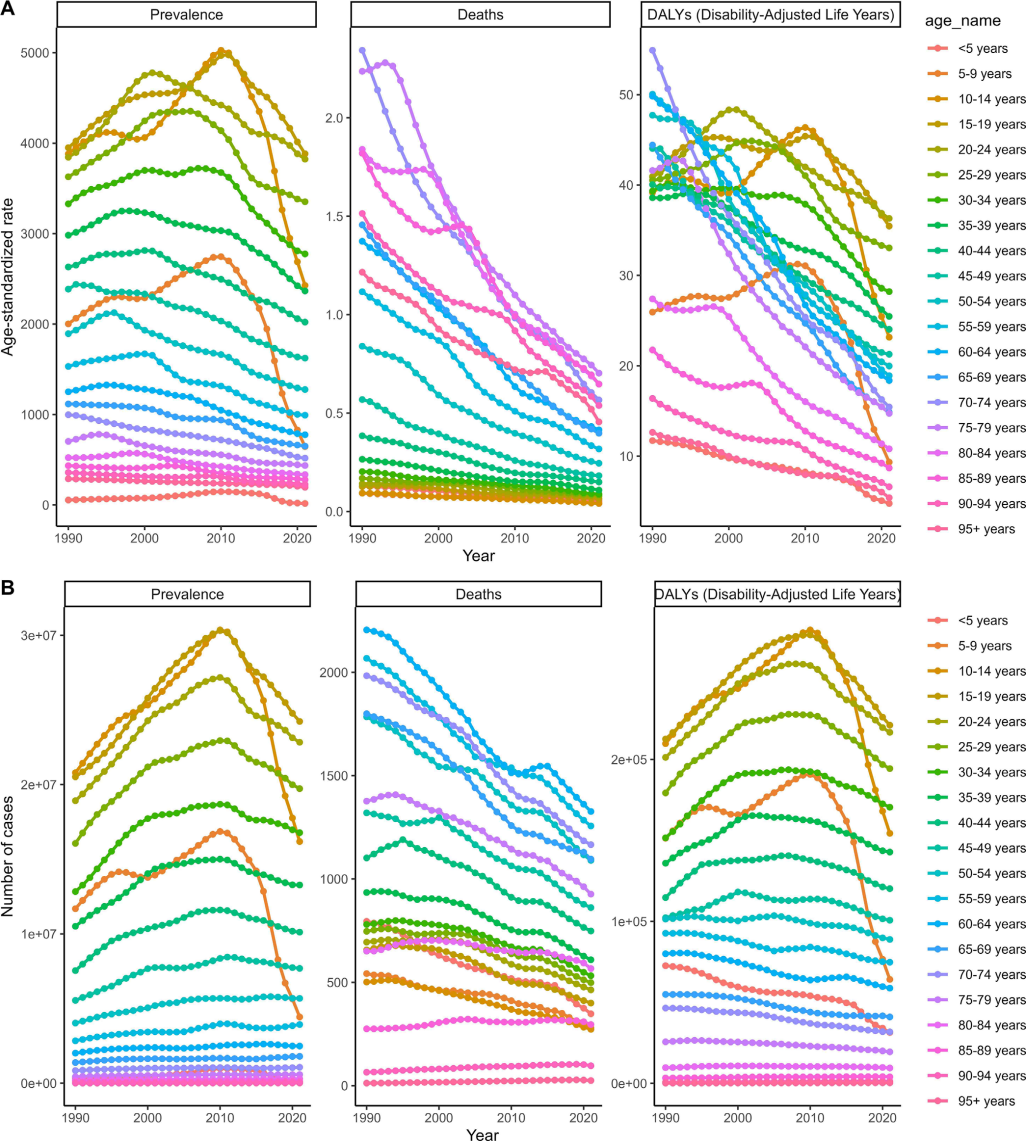
Trend for schistosomiasis in age subgroups from 1990 to 2021. (A) ASMR, ASDR, and ASPR. (B) Number of deaths, DALYs, and prevalence numbers.

**Supplementary Figure 2.**
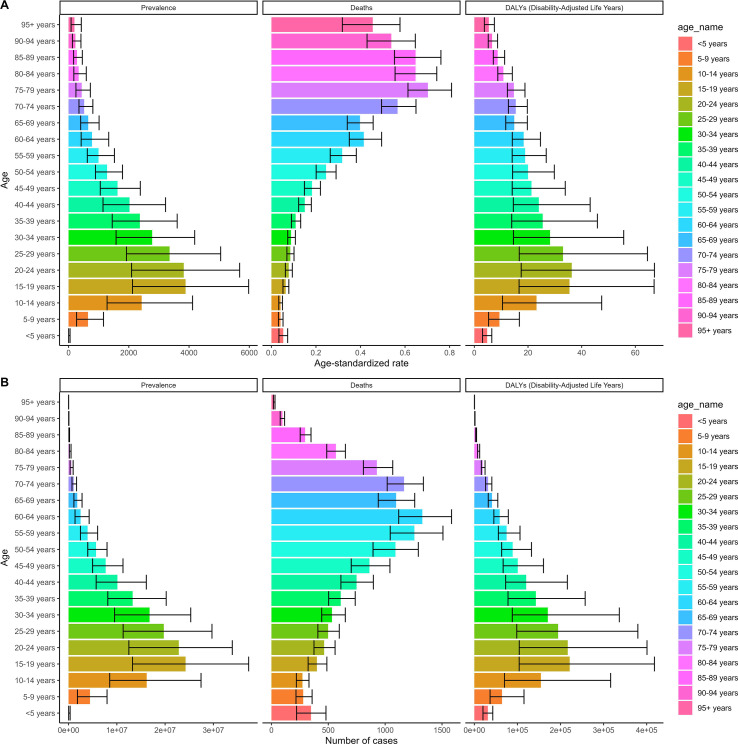
Schistosomiasis burden in age groups in 2021. (A) Age-standardized rate of deaths, DALYs, and prevalence. (B) The number of deaths, DALYs, and prevalence.

**Supplementary Figure 3.**
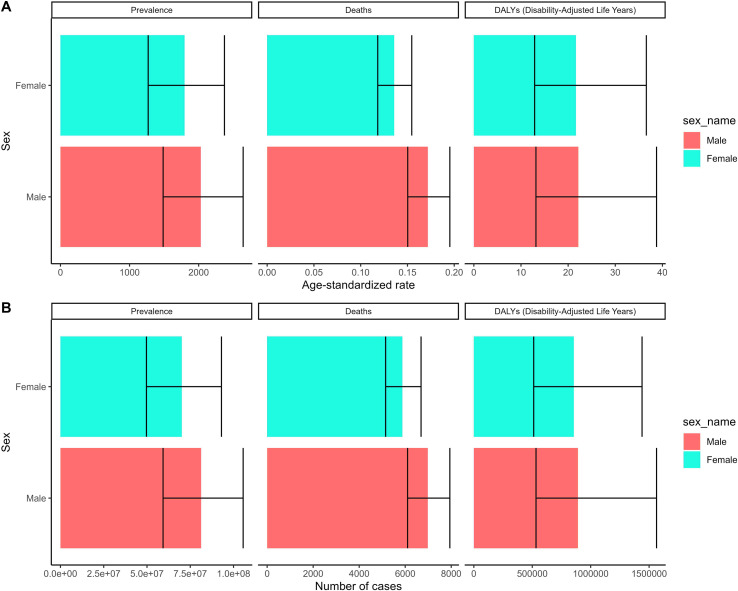
Schistosomiasis burden in sex groups in 2021. (A) Age-standardized rate of deaths, DALYs, and prevalence. (B) The number of deaths, DALYs, and prevalence.

**Supplementary Figure 4.**
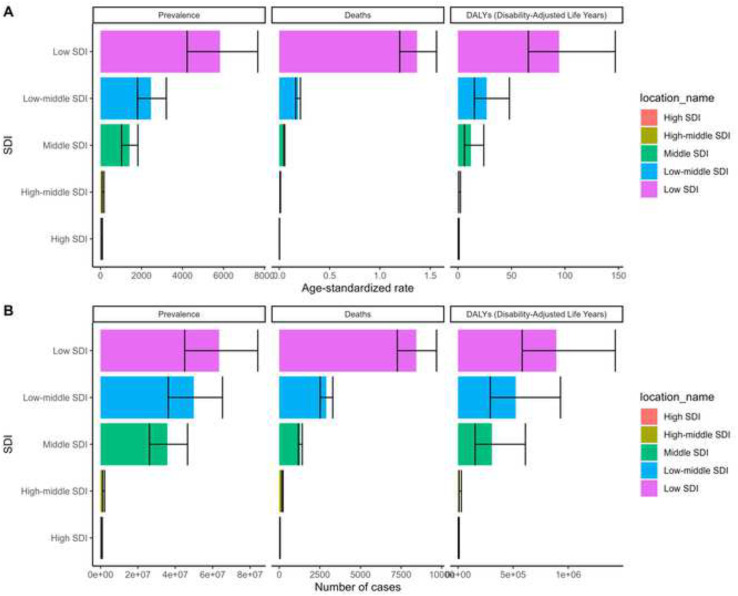
Schistosomiasis burden in the SDI group in 2021. (A) Age-standardized rate of deaths, DALYs, and prevalence. (B) The number of deaths, DALYs, and prevalence.

**Supplementary Figure 5.**
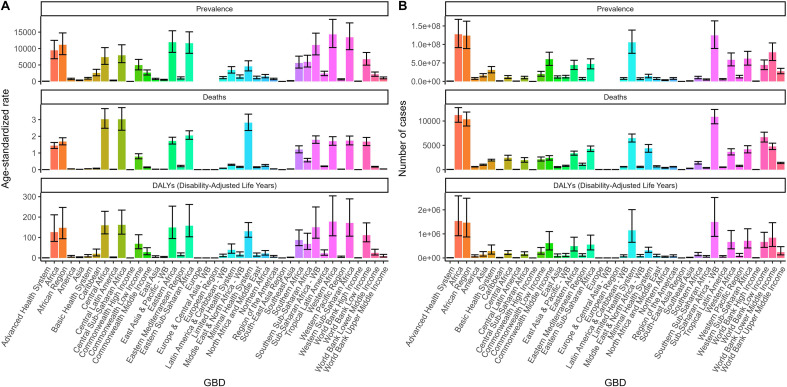
Schistosomiasis burden in the GBD region in 2021. (A) Age-standardized rates of deaths, DALYs, and prevalence. (B) The number of deaths, DALYs, and prevalence.

Further subgroup analyses of the 2021 data, as reported by the GBD 2021, revealed age-, sex-, and SDI-specific patterns. Across all age groups, the number of deaths and ASMR peaked in certain age groups before declining, while the prevalence number and ASPR peaked earlier and then gradually decreased. The DALYs and ASDR showed similar trends, peaking in younger age groups and then decreasing (Fig. 2). In terms of sex, men had higher disease burdens than women across all indicators. At the SDI level, low SDI regions had the highest disease burdens, with a gradually increasing trend from high SDI regions. Regionally, Africa and Sub-Saharan Africa – WB had the highest deaths and DALYs, respectively, while the highest prevalence was reported in multiple regions including Africa and Sub-Saharan Africa – WB (Supplementary Figs. 2–5 and Tables 1–3).

### National-level and trend analyses from 1990 to 2021 (based on GBD 2021 data)

**Supplementary Figure 6.**
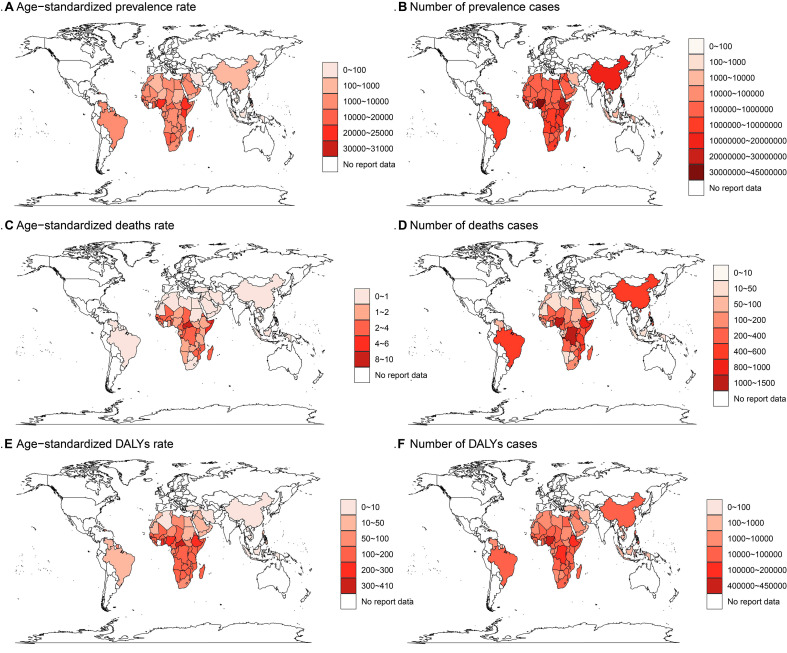
Schistosomiasis burden in countries and territories in 2021. (A) ASPR. (B) ASMR, (C) ASDR, (D) Prevalence number. (E) Number of deaths. (F) DALYs number.

At the national level, countries with reported schistosomiasis cases in 2021 were primarily located in Africa and South America, and in China. The highest ASMR, ASDR, and ASPR were reported in various countries, with Nigeria standing out for having the highest number of deaths, DALYs, and prevalence number (Supplementary Fig. 6 and Tables 1–3).

**Figure 3:**
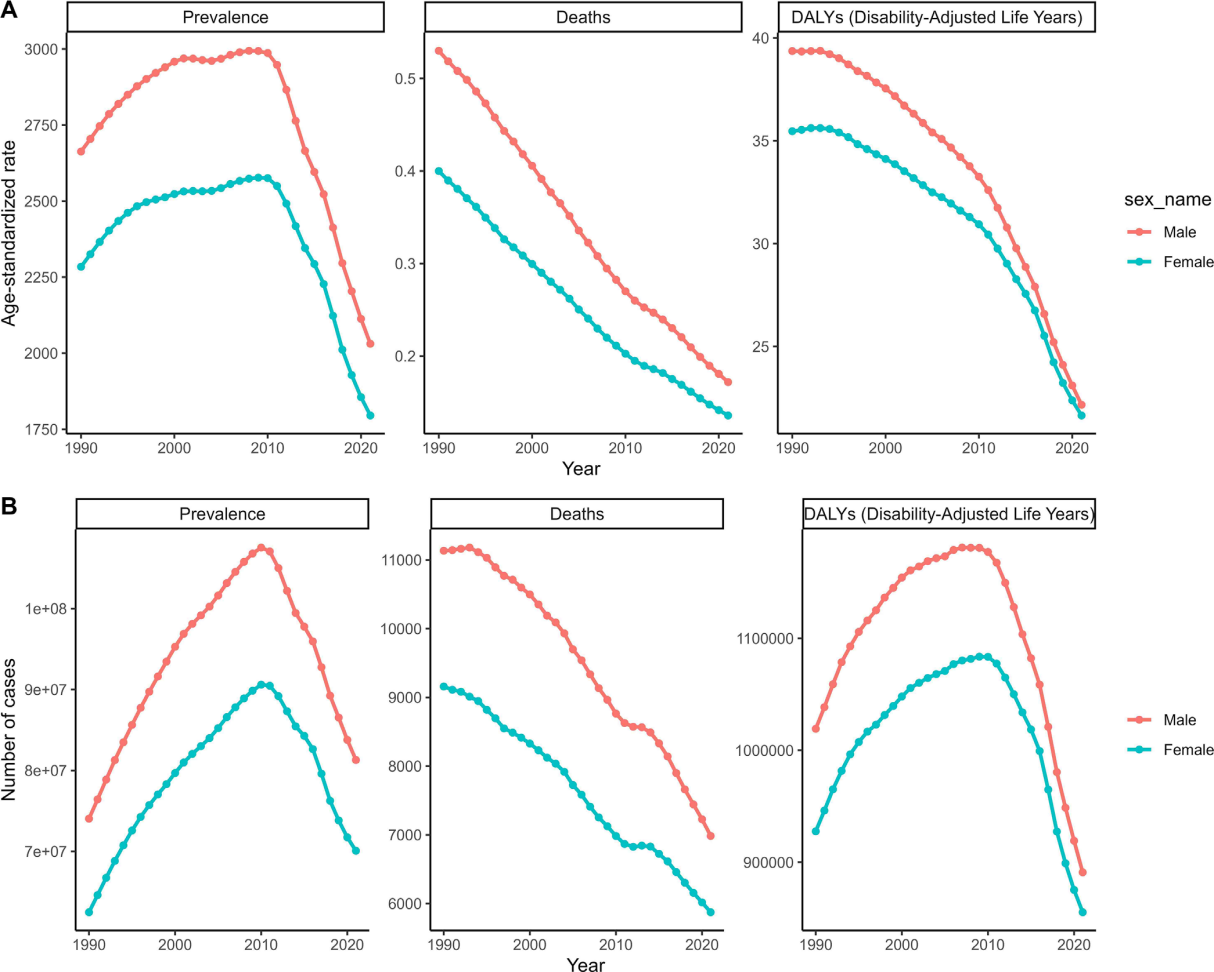
Trend for schistosomiasis in sex subgroups from 1990 to 2021. (A) ASMR, ASDR, and ASPR. (B) Number of deaths, DALYs, and prevalence numbers.

**Figure 4:**
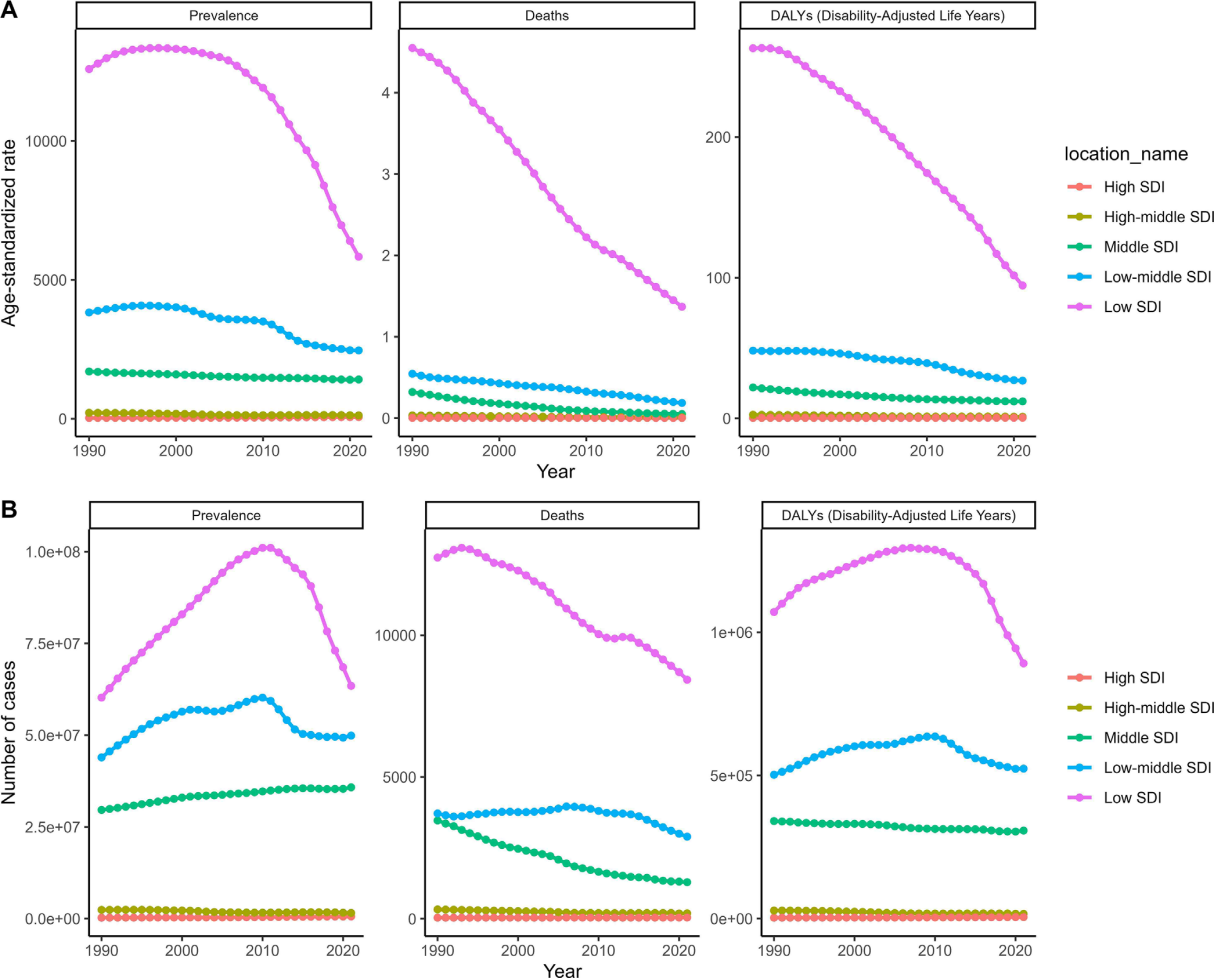
Trend for schistosomiasis in SDI subgroups from 1990 to 2021. (A) ASMR, ASDR, and ASPR. (B) Number of deaths, DALYs, and prevalence numbers.

**Figure 5:**
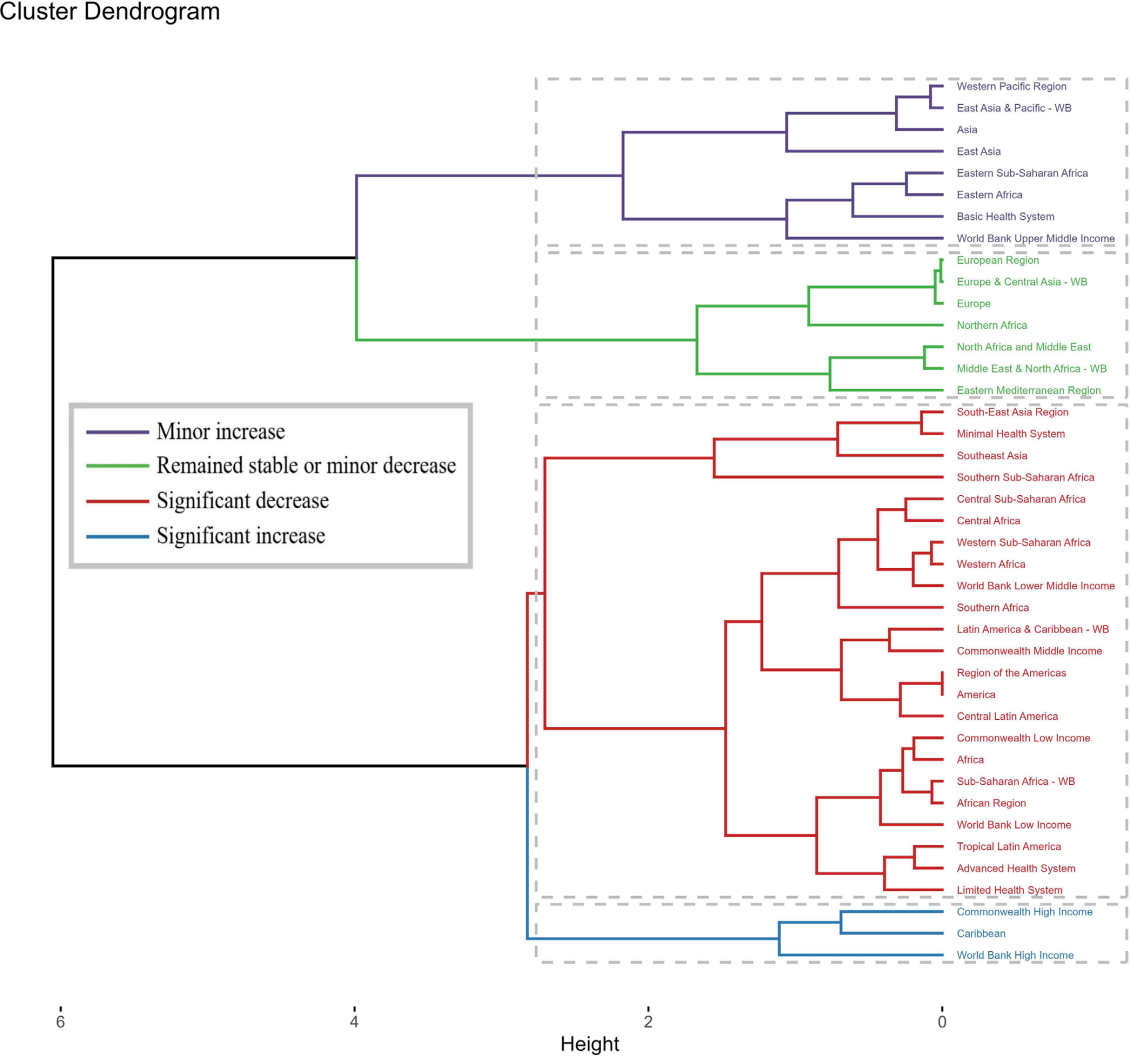
Results of cluster analysis based on the EAPC values of the schistosomiasis-related age-standardized rates for deaths, DALYs, and prevalence from 1990 to 2021.

**Figure 6:**
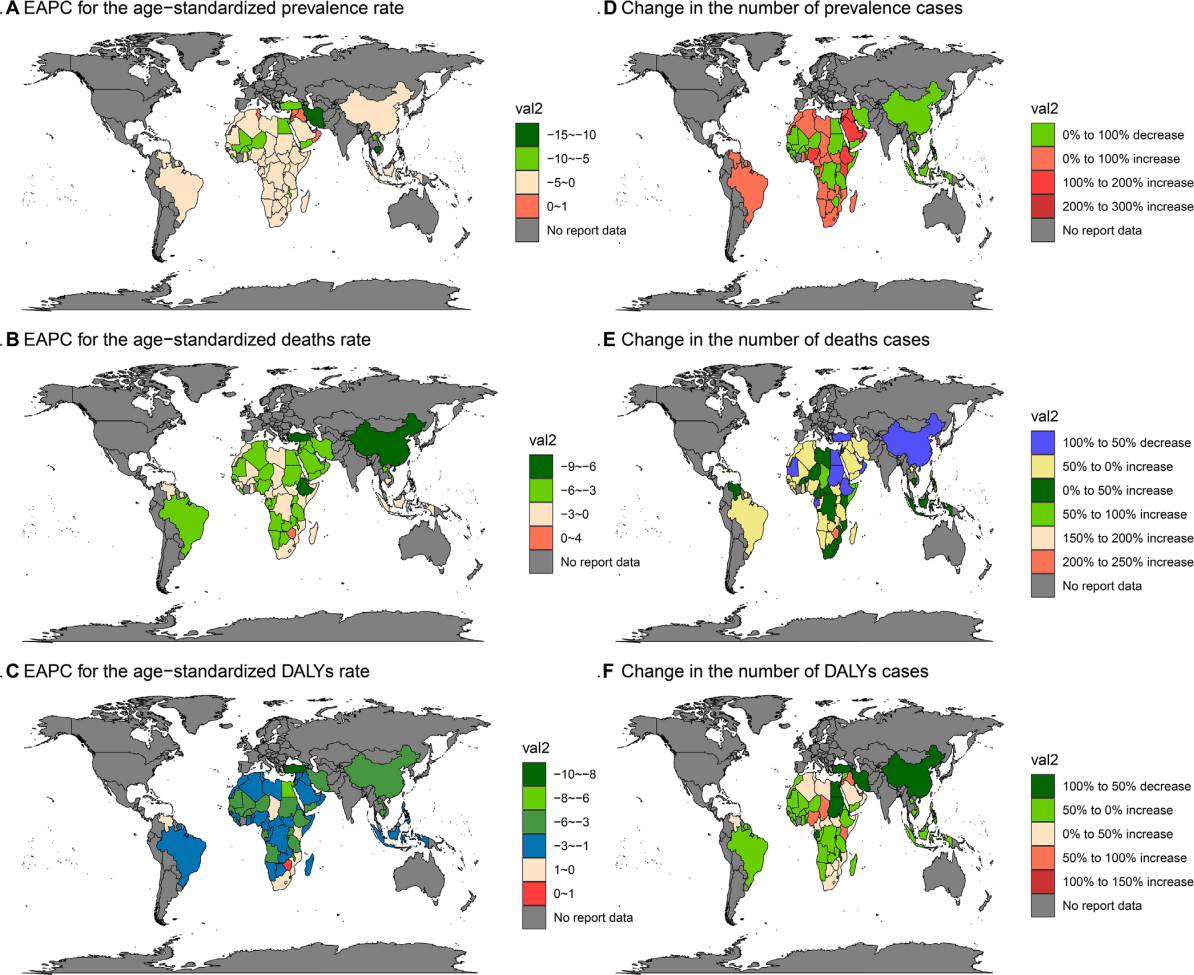
Trend for schistosomiasis in national and territories subgroups from 1990 to 2021. (A) ASPR. (B) ASMR. (C) ASDR. (D) Prevalence number. (E) Number of deaths. (F) DALYs number.

Trend analyses from 1990 to 2021, based on GBD 2021 data, revealed notable declines in deaths, ASMR, and DALYs across all age groups. For ASDR and ASPR, specific age groups experienced initial increases followed by declines, while other age groups showed more stable trends. Sex-specific and SDI-specific analyses further highlighted these trends, with significant declines observed in low SDI areas for multiple indicators (Figs. 3 and 4). Additionally, clustering analysis categorized 41 GBD regions into four trends, and world maps illustrated changes and trends in key indicators across 70 countries and regions (Figs. 5 and 6).

### Disease burden forecast of schistosomiasis

**Figure 7:**
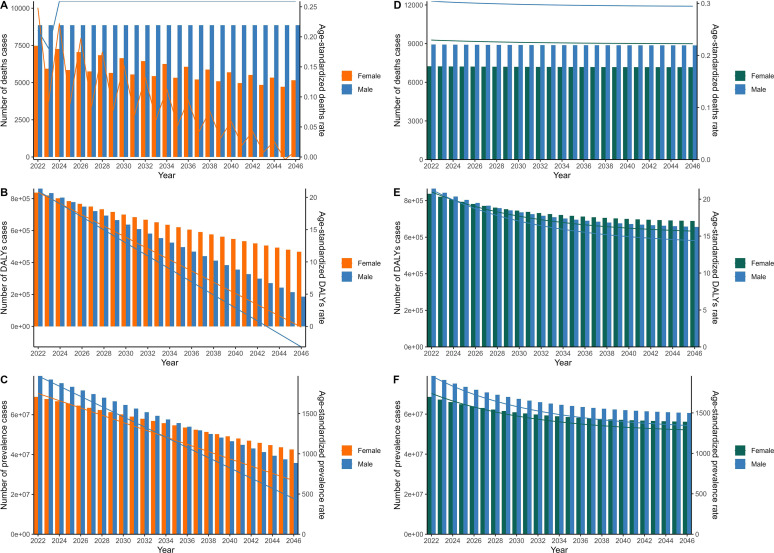
Predicted trends for global schistosomiasis in number of deaths and age-standardized mortality rate using the ARIMA model (A) and exponential smoothing model (D), predicted trends for global schistosomiasis in DALYs and age-standardized DALYs rates using the ARIMA model (B) and exponential smoothing model (E), predicted trends for global schistosomiasis in prevalence number and age-standardized prevalence rate using the ARIMA model (C) and exponential smoothing model (F).

Using time series analysis, we employed the ARIMA model and ES model to forecast the schistosomiasis burden from 2022 to 2046. Leveraging data spanning from 1990 to 2021, the ARIMA model was utilized to quantitatively predict future trends in schistosomiasis. After applying the auto.arima function for optimization, the resultant ARIMA models for various indicators were as follows: male ASMR (0, 1, 3), male age-standardized death rate (ASDR) (0, 2, 2), male ASPR (0, 2, 1), female ASMR (1, 1, 2), female ASDR (0, 2, 1), female ASPR (0, 2, 1), male death count (0, 1, 1), male DALYs (0, 2, 0), male prevalence count (0, 2, 1), female death count (1, 1, 1), female DALYs (2, 0, 1), and female prevalence count (0, 2, 1). The ARIMA model predictions indicate a decline in female deaths and ASMR fluctuations over the next 25 years, whereas male deaths and ASMR are projected to remain stable (Figs. 7A). Additionally, other indicators for both genders are anticipated to continue decreasing (Figs. 7B and 7C). To evaluate the robustness of these predictions, we further utilized an ES model to forecast the future disease burden of schistosomiasis, finding generally consistent results with those of the ARIMA model (Figs. 7D–7F).

## Discussion

This study is the first to comprehensively analyze the disease burden of schistosomiasis globally. Previous studies on this topic were mostly limited to individual countries or regions [1, 2, 9, 23, 34], and some were outdated [14, 17], so the trend and future burden of schistosomiasis disease globally were unclear. This study used the newly released GBD 2021 schistosomiasis data to estimate the global disease burden related to schistosomiasis and explored the trends and patterns of disease change from 1990 to 2021, and predicted the disease burden from 2022 to 2046. These findings may provide scientific evidence and reference for the control of schistosomiasis globally.

The results of this study show that significant progress has been made in schistosomiasis control globally over the past 30 years. Compared with 1990, five indicators, including death number, DALYs, ASMR, ASDR, and ASPR, showed a downward trend in 2021, while only the number of infected people showed an upward trend. In age subgroups, except for the ASPR trend upwards in the 15–19 age group, ASMR, ASDR, and ASPR showed a downward trend in all age groups. In the gender subgroups, except for the upward trend in prevalence number and ASPR, the other four indicators showed a downward trend. Overall, significant progress has been made in global schistosomiasis control over the past 30 years, thanks to the following factors: first, the continuous attention to and advocacy for schistosomiasis by the WHO, including a series of recommendations [8, 25]; second, the active prevention and control measures taken by disease burden regions and organizations [29, 30, 33]; and third, the widespread use of effective treatment drugs such as praziquantel [32]. These factors have greatly reduced the number of deaths, DALYs, and corresponding ASR of schistosomiasis globally. However, it is also important to note that prevalence number and the ASPR for the majority of disease burden countries have not been controlled and are on the rise.

On a global scale, both the prevalence number and the ASPR for all five SDI regions have shown an upward trend, with the lowest SDI region having the heaviest burden of schistosomiasis, but the most obvious downward trend. Although the burden of schistosomiasis in the highest SDI region is relatively light, the six indicators of death number, DALYs, prevalence number, and corresponding ASR in 2021 compared to the indicators in 1990 all show an increasing burden of disease, which may be related to the increase in global personnel flow and international trade, leading to the import of schistosomiasis into the high SDI regions [20]. Schistosomiasis mainly spreads in impoverished and barren areas with poor economic development, and it is also the region with the heaviest burden of schistosomiasis globally. Under the impetus of the WHO, various aid measures, such as the planning of schistosomiasis control programs, drug donations, and the development of diagnostic tools, have been implemented in low SDI areas, leading to a significant reduction in the burden of schistosomiasis disease [4, 8].

The research findings also show that from 1990 to 2021, the global burden of schistosomiasis disease was characterized by higher DALY years, prevalence number, and corresponding ASR in the adult group, the highest number of deaths and ASMR in the elderly group, and a relatively heavy burden in the 5–15 year school-age children groups, which may be related to factors such as poor ecological environment, lack of public health education, backward sanitation facilities, and insufficient understanding of the life cycle and causes of the disease. This result is basically consistent with the conclusions of previous studies [2, 9]. Therefore, to achieve the WHO’s global elimination of schistosomiasis public health threat target, more investment and support are needed for such low SDI areas.

The study results also show that in the 70 schistosomiasis disease burden countries and regions, more than 30 countries have seen an upward trend in disease death numbers, DALYs, and prevalence number, with most of them being African countries, such as Zimbabwe and Nigeria, as well as Oman [21]. For example, previous reports have shown that Zimbabwe implemented a national worm control project plan, providing mass praziquantel prophylactic treatment for school-age children, achieving a schistosomiasis infection rate of less than 1.00% among school-age children [22]. The results of this study also show a downward trend in schistosomiasis morbidity and ASPR in Zimbabwe in 2021, but the country’s DALYs, number of deaths, and ASMR are higher than in 1990, estimated to be due to the global population explosion over the past 30 years, coupled with the fact that the project plan mainly targeted children, so the previous reporting results cannot truly reflect the actual situation of the entire population in the country. It is therefore speculated that the situation in other schistosomiasis disease burden-heavy African countries is similar.

In this study, we employed time series analysis using both the ARIMA model and the ES model to forecast the schistosomiasis burden from 2022 to 2046. Leveraging a comprehensive dataset spanning from 1990 to 2021, our ARIMA models were tailored to predict future trends in schistosomiasis through the application of the auto.arima function for optimization. The resultant ARIMA model configurations for various indicators, including male and female ASMR, ASDR, ASPR, death counts, DALYs counts, and prevalence counts, provide a nuanced understanding of the disease’s future trajectory. Notably, our ARIMA model predictions indicate a decline in female deaths and fluctuations in female ASMR over the next 25 years, whereas male deaths and ASMR are projected to remain relatively stable. This gender-specific disparity in mortality trends may reflect differences in access to healthcare, disease management practices, or biological susceptibility to schistosomiasis [16]. Furthermore, other indicators for both genders, such as ASDR, ASPR, DALYs, and prevalence counts, are anticipated to continue decreasing. This overall decline suggests potential progress in schistosomiasis control efforts, which could be attributed to advancements in treatment, prevention strategies, and public health interventions [24].

To validate the robustness of our ARIMA model predictions, we employed an ES model, which yielded generally consistent results. This convergence of findings across different forecasting methodologies strengthens our confidence in the projected trends and highlights the potential for continued improvements in schistosomiasis control. However, it is important to acknowledge that these predictions are based on historical data and may be influenced by future changes in disease dynamics, healthcare policies, and environmental conditions. Therefore, ongoing monitoring and evaluation of schistosomiasis burden, coupled with adaptive strategies for disease control, will be crucial to ensuring the accuracy and relevance of these forecasts in guiding public health interventions.

There are several limitations in this study. First, the data in the GBD database are not real observational data, and there may be differences between the data and the actual situation when there is a lack of data from the marginal areas and specific populations. Second, the GBD database may not have covered all possible risk factors that lead to schistosomiasis. For example, the impact of infection on female reproductive organs and functions [17]. Third, there is a lack of detailed monitoring data for some key areas, especially in African countries and regions, which makes the overall burden analysis of schistosomiasis less accurate.

## Conclusions

In summary, to completely eliminate or eradicate schistosomiasis globally, especially in Africa, there are still many challenges, such as the dramatic changes in the natural environment, global warming [27], and population growth, which seriously limit the supply of health resources. Other factors include insufficient drug supply and low population coverage, no effective vaccine to date [28], inadequate information monitoring systems, and insufficient representativeness of monitoring data [31]. Early diagnostic techniques and tools need to be improved. Use of drugs for prevention and treatment is in conflict with environmental protection, such as the large-scale use of pesticides causing environmental damage, the emergence of drug resistance and other adverse reactions. The agricultural development mode is unable to effectively cut off the source of infection and transmission [26], global economic development is highly uneven, and the African region as a whole has a poor economy, social environment, and insufficient investment in public health. Therefore, a comprehensive understanding of the current global schistosomiasis epidemic situation and the characteristics of disease epidemics are the basis for exploring comprehensive prevention and control strategies, and we hope to contribute to the WHO’s goal of eliminating schistosomiasis by 2030.

## Supplementary materials

The supplementary material for this article can be found at https://www.parasite-journal.org/10.1051/parasite/2025005/olm.
